# Comparative Population Genomics of the *Borrelia burgdorferi* Species Complex Reveals High Degree of Genetic Isolation among Species and Underscores Benefits and Constraints to Studying Intra-Specific Epidemiological Processes

**DOI:** 10.1371/journal.pone.0094384

**Published:** 2014-04-10

**Authors:** Maude Jacquot, Mathieu Gonnet, Elisabeth Ferquel, David Abrial, Alexandre Claude, Patrick Gasqui, Valérie Choumet, Myriam Charras-Garrido, Martine Garnier, Benjamin Faure, Natacha Sertour, Nelly Dorr, Jocelyn De Goër, Gwenaël Vourc'h, Xavier Bailly

**Affiliations:** 1 INRA, UR346 Epidémiologie Animale, Saint Genès Champanelle, France; 2 Institut Pasteur, CNR Borrelia, Paris, France; University of Kentucky College of Medicine, United States of America

## Abstract

Lyme borreliosis, one of the most frequently contracted zoonotic diseases in the Northern Hemisphere, is caused by bacteria belonging to different genetic groups within the *Borrelia burgdorferi* species complex, which are transmitted by ticks among various wildlife reservoirs, such as small mammals and birds. These features make the *Borrelia burgdorferi* species complex an attractive biological model that can be used to study the diversification and the epidemiology of endemic bacterial pathogens. We investigated the potential of population genomic approaches to study these processes. Sixty-three strains belonging to three species within the *Borrelia burgdorferi* complex were isolated from questing ticks in Alsace (France), a region where Lyme disease is highly endemic. We first aimed to characterize the degree of genetic isolation among the species sampled. Phylogenetic and coalescent-based analyses revealed clear delineations: there was a ∼50 fold difference between intra-specific and inter-specific recombination rates. We then investigated whether the population genomic data contained information of epidemiological relevance. In phylogenies inferred using most of the genome, conspecific strains did not cluster in clades. These results raise questions about the relevance of different strategies when investigating pathogen epidemiology. For instance, here, both classical analytic approaches and phylodynamic simulations suggested that population sizes and migration rates were higher in *B. garinii* populations, which are normally associated with birds, than in *B. burgdorferi* s.s. populations. The phylogenetic analyses of the infection-related *ospC* gene and its flanking region provided additional support for this finding. Traces of recombination among the *B. burgdorferi* s.s. lineages and lineages associated with small mammals were found, suggesting that they shared the same hosts. Altogether, these results provide baseline evidence that can be used to formulate hypotheses regarding the host range of *B. burgdorferi* lineages based on population genomic data.

## Introduction

Zoonotic diseases caused by pathogens that are transmitted among different host species represent an emergent threat for human health [1]. However, the study of these pathosystems is hampered by their complexity, as each pathogen may have multiple potential reservoirs. In vector-borne systems, in which infected vectors feed on and transmit the pathogen to several hosts, it is possible to characterize pathogen diversity using population genomic studies. These studies, which use information obtained from the genome of pathogens isolated from questing vectors, offer researchers the opportunity to indirectly study the spread of pathogens within and among host communities. However, genetic information can be affected by many processes, and untangling various lines of evidence in order to obtain a coherent picture of the evolutionary history of a population represents a significant challenge for population genomic studies [2].

In particular, the selective constraints on pathogen genomes can be highly heterogeneous. For example, infection-related genes are expected to be affected by disruptive and/or negative frequency-dependent selection due to the molecular interactions occurring among pathogens, hosts, and the hosts' immune systems. Therefore, within bacterial lineages associated with similar hosts these genes would be predicted to share similar features due to host-driven selective sweeps, DNA exchange by recombination, or convergent evolution. Patterns of diversity in these genes could thus potentially reveal which lineages infect overlapping host communities. Alternatively, housekeeping genes, which are involved in basic cell cycle and metabolic functions, are mostly subject to purifying selective pressures [3]. They are more likely to contain relevant information about gene flow among pathogen populations and other demographic events, especially if pathogen lineages have had the opportunity to recombine [4]. Indeed, most regions of the genome should not be affected by host driven selection if the recombination rate is sufficiently high [5], which is an advantage for researchers examining demographic and/or epidemiological processes. Infection-related genes and other genome regions can thus provide complementary information on bacterial transmission. One of the main challenges of population genomic approaches is to integrate hypotheses about patterns of diversity that are observed at the scale of individual genes or genome regions into evolutionary scenarios that are coherent at the level of the whole genome.

Using appropriate genome regions, analyses of demographic and epidemiological processes from population genomic data rely on models that describe both the way pathogens spread within and among susceptible individuals, as well as processes that govern genome evolution. These two aspects have historically been investigated independently in the literature. On the one hand, the spread of pathogens has often been studied by epidemiologists using population dynamic models that take into account complex infection and transmission processes [6], but most of the time neglect the diversity of pathogens. On the other hand, the evolution of genomes has been studied using tools of population genetics that often assume basic demographic hypotheses, as in the Wright-Fisher [7] or the Moran [8] model. The increasing prevalence of molecular epidemiology studies highlights the need to close the gap between these two approaches, a feature that defines the burgeoning field of phylodynamics. While phylodynamic approaches have produced promising results in the study of epidemics of fast-evolving viruses, their applicability to endemic, slow-evolving, bacterial pathogens remains to be assessed [9]. A particular challenge for the use of phylodynamic models is that genomic polymorphisms represent the footprints of processes that have occurred across multiple geographic and/or time scales. For example, phylogeographical studies have shown that a population's history plays a major role in shaping its current diversity patterns. Large-scale patterns, such as the occurrence of major clades within a species can be due to ancestral differentiation in independent geographic locations (called refugia), while the distribution of diversity on a smaller scale is subsequently shaped by more recent colonization and migration events [10]. The picture is even more complex in multi-host pathogen systems, in which major clades can emerge either from isolated geographic regions where the pathogen circulates or from independent reservoir hosts as defined by Haydon *et al.* [11]. Then, the diversity that is maintained in a given reservoir at endemic equilibrium would depend not only on epidemiological parameters such as the number of infected hosts and transmission parameters, but also on the processes that generate sequence diversity in that population [12].

In core regions of bacterial genomes, sequence diversity emerges through both mutation and homologous recombination. Twenty years ago, Maynard Smith *et al.* revealed the impact of recombination on the evolution of bacterial pathogens, which can be hidden by differences in the reproductive success of genotypes [13]. In the so-called epidemic population structure, the uneven frequencies of recombinant lineages lead to high statistical associations among genotypes at different loci, i.e. linkage disequilibrium. By selecting a subsample of unique multilocus genotypes within a population, Maynard Smith and colleagues were able to more easily identify statistical evidence for recombination. Although still relevant, this sub-sampling approach has major drawbacks, as it is difficult to quantitatively study the processes that shape the distribution of diversity in subsamples that have been defined by genetic criteria *a priori* [14]. Therefore, studies of pathogen diversity commonly use hierarchical sampling. Factors that structure diversity, such as bacterial taxonomy or sampling location, are defined *a priori*, and a random sample of strains is obtained for each selected factor [15]. Then, the accuracy of species delineations (or whichever structuring factor is used) can be evaluated *a posteriori* with regard to the observed patterns of polymorphism before fitting more complex evolutionary and epidemic models to the genomic data.

To assess the potential of population genomic approaches in the study of multi-host zoonotic disease, we chose the *Borrelia burgdorferi* species complex as our model system. This species complex includes the bacteria that cause Lyme borreliosis, one of the most common vector-borne diseases in the Northern Hemisphere. In Europe, most human cases are caused by *Borrelia afzelii*, *Borrelia burgdorferi* sensu stricto (s.s.), and *Borrelia garinii* [16], which are transmitted by the tick *Ixodes ricinus* among different reservoir species. The ticks can become infected during their first blood meal, which occurs before they molt from larvae into nymphs. They can also acquire and/or transmit bacteria during their other blood meal(s), which occur before the molt from nymph to adult (all ticks) and before laying eggs (females only).

Bacteria within the *B. burgdorferi* complex do not share a common, fixed set of host species (referred to hereafter as host range) [17]. Phylogenetic analysis of multi-locus sequence typing (MLST) data suggests that similar host ranges have evolved several times within this complex, so that bacteria associated with similar hosts are not clustered in clades, but rather, dispersed throughout the whole phylogeny [18]. *Borrelia garinii* and *Borrelia valaisiana*, which are only distantly related to each other, both infect birds [19]–[22], whereas *B. afzelii*, *Borrelia spielmanii*, and *Borrelia bavariensis*, which do not cluster together in phylogenies, infect small mammals [23]–[25]. Furthermore, the host range of *B. burgdorferi* s.s. appears to be broader than that of most *Borrelia*, as this species has been identified in both small mammals and birds [26]–[28]. However, despite the large number of host shifts that have occurred through the course of the evolution of this species complex, the influence of mutation and recombination events on these bacteria's ability to adapt to new hosts is poorly understood [29].

Different approaches have been used to investigate the influence of host communities on the diversity of the *B. burgdorferi* species complex. From a population genetic point of view, differences in the population size and the migration potential of reservoir species have been shown to influence MLST diversity patterns of these bacterial species at a continental scale [30], but there is a lack of information at smaller geographical scales. From an epidemiological point of view, a statistical model has been used to estimate the respective contributions of different host species to the infection of ticks by genotypes of *B. burgdorferi* s.s. in the USA [31]. This approach takes advantage of the associations found among genotypes of the *B. burgdorferi* species complex obtained from different populations and host species at the infection-related gene *ospC* [32], [33].

The development of genetic and genomic tools has allowed the in-depth investigation of genes involved in infection phenotypes within the *B. burgdorferi* species complex [34]. A recent study of synonymous and non synonymous mutation rates from multiple genomes has highlighted a potential role for lipoproteins-encoding genes in the adaptation of the *B. burgdorferi* species complex to hosts [35]. The same sample of genomes, which was predominantly obtained from strains of *B. burgdorferi* s.s., was also used to investigate evolutionary processes within this species [36], although inter-specific patterns were described only superficially. While the focus of this study was to investigate recombination patterns and selective constraints within *B. burgdorferi* s.s., the selected isolates were chosen in order to capture a maximum of genetic diversity [37]. However, as described above, this type of sampling could reveal a different genetic structure compared to a random regional sampling.

Here, we investigated the genomic diversity of strains of *B. burgdorferi* s.s., *B. garinii,* and *B. afzelii* isolated from questing ticks in France, in a region where Lyme borreliosis is highly endemic. Using a hierarchical sampling scheme at a regional scale, we aimed to investigate the genetic diversity within and isolation among species of the complex. We also evaluated multiple models, involving both evolutionary and epidemiological constraints, and examined the information obtained from them to understand the forces that have shaped diversity within this species complex. This lead to different hypotheses regarding the evolution and the epidemiology of these bacteria. The data gathered here are then discussed in the context of what is currently known about the ecology and evolution of these vector-borne pathogens.

## Materials and Methods

### Ethics statement

**Figure 1:**
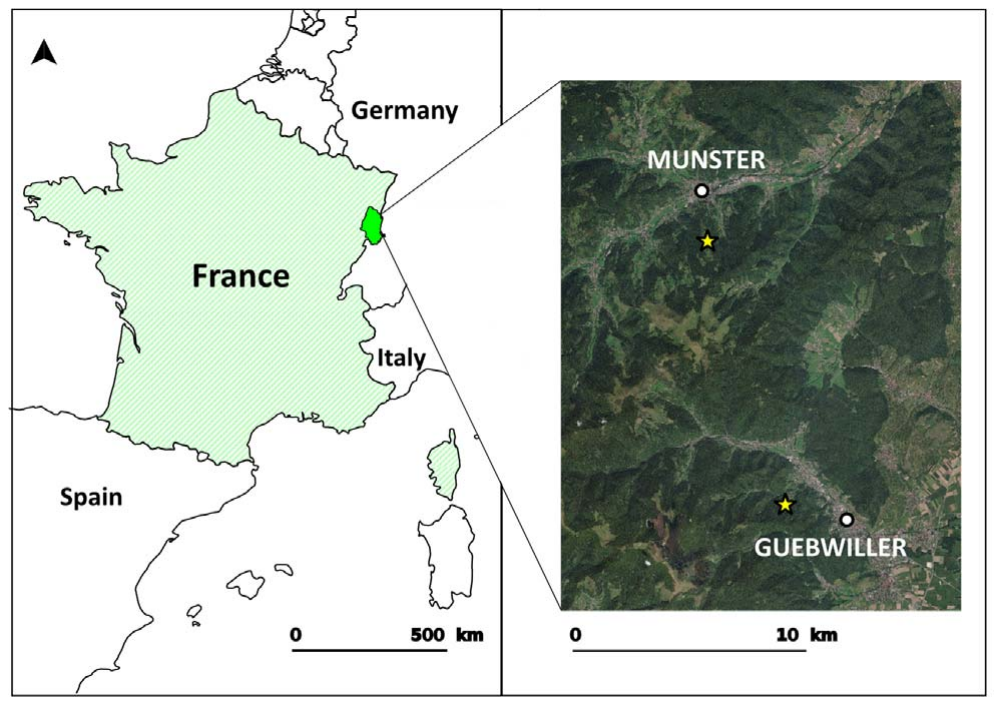
Map of the sampling sites. Tick sampling sites (yellow stars) were located near the towns of Munster and Guebwiller in northeastern France. The data used to construct the map were obtained from BD-Ortho in 2011 (IGN; National Institute of Geographic and Forest Information, Paris, France).

Isolates of the *B. burgdorferi* species complex were recovered from questing nymphs and adults of *I. ricinus* that were sampled at two sites largely covered by dense and continuous forests; these sites were located near the towns of Munster and Guebwiller, in Alsace (France) [38] (Figure 1). No specific permission is required by French law to sample ticks and to perform field studies in these locations. Our study did not involve any endangered or protected species.

### Bacterial strains and sequencing procedures

Ticks were collected at each site in two consecutive years (2003 and 2004) by dragging a large piece of cotton fabric across the vegetation and leaf litter [38]. Ticks were then kept alive in individual tubes until they could be processed. Bacterial isolation was performed by incubating ticks individually at 32°C in 5 ml of BSK-H medium (Sigma-Aldrich, St. Louis, USA) without antibiotics for 8 weeks. Bacterial growth was checked weekly via dark field microscopy. Cultures that showed evidence of spirochete growth were maintained until they reached high densities. After that PCR-RFLP was used to assign each culture to a *Borrelia* species [39]. Lastly, each culture was supplemented with 60% glycerol in an equal amount to that of the culture and kept at −80°C, pending further analyses.

From the different isolates, we chose 63 strains, to be sequenced. As we aimed i)to study species delineations within the *B. burgdorferi* complex, ii) to examine the distribution of diversity within sympatric species of the complex at a regional scale, and iii) to compare the genetic structures of different bacterial species, we selected a set of strains that included 25 genotypes of *B. burgdorferi* s.s., 34 genotypes of *B. garinii*, and 4 genotypes of *B. afzelii*. Within each species, genotypes were randomly selected. The size of the three samples was determined by the rate of success of the isolation procedure rather than by the observed frequency of the different species within ticks. Moreover, we selected isolates with the goal of having a similar number of strains for each sampling site within each species. We also tried to select strains that were sampled during the same year in order to limit confounding factors: all *B. garinii* and *B. afzelii* strains were isolated in 2004, whereas an equal number of *B. burgdorferi* s.s. strains were isolated in 2003 and in 2004 (Table S1).

To obtain enough material for sequencing, bacteria were iteratively cultured using 50 ml of BSK-H medium (Sigma-Aldrich) until high bacterial densities were reached. All strains underwent fewer than 15 passages from tick incubation to DNA extraction in order to limit the possibility of plasmid loss. Independent DNA extractions were performed using the DNeasy Blood & Tissue Kit (Qiagen, Venlo, Netherlands). The quantity of DNA after extraction was measured by spectrophotometry at 260 and 280 nm (Nanodrop, Thermo Fisher Scientific, Waltham, USA), and DNA extractions were sent to Genoscreen (Lille, France) to be tagged with standard multiplex identifiers (MID, Roche, Basel, Switzerland). Samples were then mixed to prepare libraries, which were distributed among three-quarters of a GS FLX Titanium (Roche) sequencing plate.

In order to analyze sequence data, raw reads were first mapped on reference sequences. Contigs were aligned with reference genomes. The genetic structure of the sample was studied using single nucleotide polymorphisms (SNPs)-based analyses at intra- and inter-specific levels. Additionally, phylogenetic analyses were performed at the same scales. Loci showing atypical polymorphism patterns were identified using allelic spectrum-based approaches and the functions they encode were analyzed. Finally, the results of SNP-based analyses on the chromosome, which revealed homogeneous polymorphism patterns, were used to fit a long term coalescent based model and a basic, shorter term, phylodynamic model. These different steps are described in details below.

### Reconstruction of genetic sequences

To obtain robust data, each sequence read was mapped independently onto reference sequences of *B. burgdorferi* s.s., *B. bavariensis*/*B. garinii*, and *B. afzelii*. For each of the three mappings, reference sequences of the chromosome, the circular plasmid cp26, and the linear plasmid lp54 were chosen from public databases (Table S2). We focused on the chromosome and the plasmids cp26 and lp54 because the gene content and the synteny of these replication units are relatively conserved among strains of the *B. burgdorferi* species complex [35]. Other replication units tend to show more polymorphism, both in terms of gene copy number and in term of gene arrangement. We did not include them in this study because of the problems they raised with sequence assembly and the specific analyses they required. To ensure accurate mapping in divergent genomic regions while maintaining sufficient stringency for subsequent analyses, we used gsMapper software (Roche) parameterized as follows: the length of seeds used to anchor alignments was fixed to 10 base pairs (bp) and the identification of three seeds per sequence was required for alignment analysis. Identity thresholds were fixed at 60 bp and 60% identity. The results of mapping onto a given replicon of a given species were stored if more than 90% of the reference sequence was covered. Then, a strict consensus sequence was generated for each replication unit of each bacterial isolate from the alignment of the stored mapping results. This was performed using a program we developed in Pascal; unless otherwise indicated, programs developed in Pascal were used for all analyses described below.

Finally, to compare our genomic data to published sequences (Table S3), we constructed multiple alignments for each of the three studied replication units. ProgressiveMauve software [40] was used to define homologous sequence regions using default parameters, and local alignments were refined using Muscle software [41] on contiguous windows of 1 kilobase pairs (kb) in length.

### Single-nucleotide polymorphism analyses

SNPs were identified using multiple alignments. To explore the population structure of *B. burgdorferi* s.s. and *B. garinii*, a set of SNPs was for each species that included all polymorphic sites in the chromosome that were identified in at least 90% of strains. The two sets of SNPs were analyzed separately using the program Structure v2.3.4 to identify potential populations and explore their degree of admixture [42]. This method has been developed to estimate allele frequencies in an user-defined number of populations and to assign individuals to these populations based on a Markov Chain Monte Carlo (MCMC) scheme. For each species, we performed analyses assuming correlations among linked loci and allowing admixture among potential populations. We investigated models with K, the number of populations, ranging from 1 to 15. For each value of K, five different runs were performed in which the MCMC algorithm was run for a 25,000-iteration burn-in step and followed by 25,000 further iterations. The most appropriate K values were chosen by taking into account the observed likelihood and the reproducibility of results.

In order to limit the impact of base-calling errors (which should be independent among individuals) and to obtain sufficient statistical power for subsequent analyses, we selected SNPs that: i) were present in all individual strains and ii) had at least two alleles with frequencies higher than 10%. Differentiation measures reflect the genetic variability among groups of individuals relative to the variability of a whole sample [43]. In order to explore the distribution of genetic variability, we chose to measure differentiation, using *H*_ST_ values [44]. *H*_ST_ is a multiallelic numerical analog of Wright's *F*_ST_ [45]_,_ and is obtained using the formula *H*_ST_ = 1-*H*_S_/*H*_T_, where *H*_S_ and *H*_T_ represent Nei diversity indices within and among populations, respectively. We calculated *H*_ST_ for selected SNPs at different levels of genetic resolution: i) among species of the complex, to assess their genetic isolation from each other and ultimately identify evidence of inter-specific homologous recombination; and ii) within studied species and between sampling sites, in order to investigate geographic isolation and identify genomic regions evolving under particular selective pressures [46]. As no evidence of genetic differentiation was observed between strains of *B. burgdorferi* s.s. sampled in 2003 and those sampled in 2004 (data not shown), we did not consider this potential structuring factor in the analyses described above. The significance of the *H*_ST_ values was tested with a Monte-Carlo approach (*p* = 0.05): for each SNP, 1000 simulated datasets that distributed individual genotypes at random among populations were created. Additionally, we compared the distribution of *H*_ST_ values in bacterial populations of *B. burgdorferi* s.s. and *B. garinii* that were sampled in different locations using a Wilcoxon rank-sum test with continuity correction, performed in R [47].

As the distribution of *H*_ST_ values depends on the genetic linkage between SNPs, we investigated the impact of homologous recombination on the genetic diversity of the studied strains. Standardized linkage disequilibrium measures between pairs of SNPs were obtained using the D' statistic [48], and the approach proposed by Hedrick [49] to summarize linkage disequilibrium among different allelic combinations. D' values were computed for each pair of SNPs within the following groups: i) all strains, ii) all strains assigned to the same species, and iii) conspecific strains isolated from both of our sampling sites in Alsace. For each species, we then explored the relationship between D' values and physical distance.

### Phylogeny-based analyses

To conduct detailed analyses of the phylogenetic relationships among genotypes using the alignments of the three replication units, contiguous 1-kb-long windows were created. We decided not to work at the gene level in order to standardize the amount of phylogenetic information contained in each unit. A phylogenetic search that applied a maximum-likelihood approach using PhyML software [50], was performed on each window. The most appropriate model of evolution was chosen for each alignment based on the Akaike Information Criterion (AIC) [51] using the APE library in R [52]. For each window, the maximum-likelihood tree was screened to assess whether different species or groups of species were monophyletic. This analysis provided information about which genome regions supported the consensus phylogeny and, conversely, which genome regions were potentially affected by interspecific recombination or other evolutionary events.

To illustrate global genetic relationships within and among species, phylogenetic networks, based on chromosomal sequences of sampled genotypes and reference genomes, were constructed with SplitsTree 4 software [53] using the Neighbor-Net method [54]. To generate these networks, we first created an alignment of chromosome sequences, and then used this alignment to compute a distance matrix in Paup* 4.0 b10 [55] using a GTR+I+G model [56], [57] with the following settings: the substitution rate matrix was estimated via maximum likelihood assuming empirical nucleotide frequencies, while the proportion of invariable sites and the shape parameter of the gamma distribution were fixed at the respective mean values that were obtained from the maximum likelihood phylogenetic analyses of the 1-kb-long windows described above.

Next, we assessed whether the percentage of windows in which a given species was not monophyletic could be explained not by recombination but simply by a lack of resolution. With this aim, we simulated 300 alignments of 63 sequences of 1000 kb according to i) the GTR+I+G model described above and ii) a neighbor-joining tree [58] obtained from the average distance matrix described in the previous paragraph and based on the appropriate model of sequence evolution that described divergence patterns among the studied genomes. For each simulated alignment, 1,000 1-kb-long contiguous windows were delineated and a maximum likelihood phylogeny based on the assumed model of sequence evolution was obtained for each window. For each phylogeny, the monophyly of species was assessed as described for observed data. Afterwards, the distribution of the percentage of windows in which species were not monophyletic within the simulated alignments was compared to that obtained from observed data. Simulations and phylogenies were generated using the Bio++ C++ library [59].

### Further alignment-based analyses

To describe diversity within each of the three species, two statistics were computed using contiguous 1-kb windows of alignments of each replicon for each species: i) Watterson's θ_S_ which is based on the number of segregating sites along the alignment [60], and ii) Tajima's θ_π_, which is based on the average divergence among samples [61]. Furthermore, to obtain insight into deviation from demographic equilibrium and selective neutrality within the *B. burgdorferi* species complex, values of Tajima's D [62] were calculated for the 1-kb windows within each replication unit for each species. Again, we chose not to work at the gene level to standardize the amount of available information among analyses; annotated genes in genomes of the *B. burgdorferi* species complex vary widely in length, with some too short to provide an adequate amount of mutation for this type of analysis. Gapped sites were not considered, as in Tajima's original publication. Tajima's D statistic measures the difference between the two estimators of θ previously described, standardized by the variance of this difference. Under the neutral evolutionary model, which assumes that polymorphisms segregate at mutation–drift equilibrium, Tajima's D is expected to be null. Positive Tajima's D value can be induced by balancing selection, due to the maintenance of highly divergent variants. Conversely, negative Tajima's D values can be the result of purifying selection or a selective sweep, which both result in an excess of weakly divergent alleles. However, these two patterns (positive or negative) may alternatively be due to demographical/epidemiological processes that affect the whole genome. Trying to distinguish between potential whole-genome versus gene-region-specific process, we looked for windows within each species' sequence that were characterized by a highly structured pattern of diversity. These genome regions might be evolving under host-driven selective pressures. Towards this end, we first obtained the distribution containing Tajima's D values for every window of chromosomal data for each studied species. As Tajima's D values were, on average, negative (see results), standard tests that identify deviations from neutrality did not provide relevant information. We thus studied chromosomal Tajima's D values and defined a threshold that distinguished relatively high measures within each species, based on the 95^th^ percentile of the distribution of Tajima's D values within that species. Then, we identified and selected the windows in the three replication units that had Tajima's D values that were higher than the defined thresholds.

Genes that overlapped the selected windows were identified and compared among the different species, and we created a non-redundant list containing the selected genes. The function and the cellular localization of the proteins encoded by the selected genes were studied using the online pipeline SLEP [63]. The distribution of genes among the different functional categories was compared to the results obtained from the total proteome of the three studied replication units.

Differentiation measures, Tajima's D values, and linkage disequilibrium patterns revealed that a 4-kb region around the *ospC* gene on the cp26 plasmid was characterized by a peculiar polymorphism pattern. To illustrate the amount of incongruent phylogenetic information in *ospC* sequences and flanking regions, Neighbor-Net networks, based on uncorrected p-distances, were obtained from alignments of the *ospC* gene, the 2,000 bp before and after the gene, and regions located further upstream and downstream.

### Long-term coalescent based model

As described in the introduction, genome sequences contain evidences of processes that occur at different time scale. In order to study the long-term processes that have shaped the diversity of the *B. burgdorferi* species complex, we developed a coalescent model to investigate the divergence between *B. burgdorferi* s.s. and *B. garinii*. This model used species-specific properties to simulate sequence datasets and employed Approximate Bayesian Computations (ABC) [64] to obtain inferences regarding parameters of interest.

The original coalescent model can be defined as a Markov process describing how, assuming a Wright-Fisher or Moran model of genetic variation, sampled individuals would share a common ancestor (coalesce) as one goes back in time [65]. A central result of this is that the number of generations that one must go back in time to the next common ancestor shared by one pair of samples in a population, i.e. a coalescence event, follows an exponential distribution, the shape of which depends on both the number of samples included in the analysis and the effective population size. Using an iterative procedure, the properties of an entire genealogy of samples can be recovered and the genealogy can then be used to simulate sequence data.

In its simplest form, the coalescent model assumes a lack of recombination and a lack of population structure; however the model has been extended to allow for such evolutionary events as homologous recombination [66] or population subdivision [67]. For example, to include recombination in the model, the exponential distribution is modified so that it describes the time before either a coalescence event or a recombination event. Similarly, to take population subdivision into account population, the exponential distribution is modified so that: i) it describes the time interval to previous coalescence event in each population, and ii) the population size of the two populations can differ, using scaling factors.

The model we developed for this study assumes two steps. During the first step, two populations, representing *B. burgdorferi* and *B. garinii*, are simulated, characterized by constant effective population sizes of c_1_*N and c_2_*N, respectively. Coalescence events are only possible within populations, and unidirectional homologous recombination is allowed both within and between populations. In each generation, for each individual, any sequence site in a given population is susceptible to intra-population homologous recombination at respective rates of *r_intra1_* and *r_intra2_*. Similarly, recombination events can occur between individuals belonging to different populations at rates *r_inter1_* and *r_inter2_*. After M * (c_1_*N+c_2_*N) generations during which the two populations evolve independently, a new step is initiated. The two populations merge into a single ancestral population, with effective population size c_3_*N and intra-population recombination rate *r_intra3_*. As it is impossible for the populations to coalesce into a single common ancestor if the probability of recombination is higher than the probability of coalescence, if the common ancestor as not been reached after (*M*+*S*) *(c_1_*N+c_2_*N) generations, recombination is stopped. Once a complete genealogy is obtained, mutations are added along branches at a rate *μ* per site per generation according to the Jukes–Cantor model [68].

Various nested models were used to simulate alignments of 10,000-bp sequences that included 23 samples for the first population (*B. burgdorferi* s.s.) and 32 samples for the second (*B. garinii*). In our initial model (M0), all the variables described above were assumed to be independent. In model M1 and all subsequent models, we assumed *r_inter1_* = *r_inter2_*. In models M2 and M3, we assumed, respectively, *c_1_* = *c_2_ = c_3_* and *r_intra1_* = *r_intra2_* = *r_intra3_*, while the M4 model incorporated both of these assumptions. We finally investigated an M5 model that assumed *r_inter1_* = *r_inter2_* = *r_intra1_* = *r_intra2_* = *r_intra3_* and *c_1_* = *c_2_ = c_3_*. After exploring model behavior, we performed 50,000 simulations for each model, fixing the parameters as follows to shorten computation time: *N* = 1e+09, *μ* = 1e-07 and *S* = 5. Based on the relationship we observed between linkage disequilibrium and physical distance on the genome (see results), we assumed a fixed recombination fragment length of 500 bp, a length that is consistent with current hypotheses about recombination track lengths [34]. For each simulation, we sampled values for the different parameters of interest in uniform distributions ranging from 0 to 1e-08 for *r_inter_,* from 0 to 8e-07 for *r_intra_,* from 5e-07 to 2e-05 for values of *c*, and from 1 to 35 for values of *M*.

From each simulation, we computed a set of summary statistics. In order to fit the diversity of simulated populations to the observed dataset, we identified SNPs from simulated alignments as previously described. To calibrate diversity patterns within populations, we computed the density of SNPs along the simulated sequences and the average Nei diversity indices for the selected SNPs. We also measured linkage disequilibrium between pairs of SNPs using the D' statistic within and among populations to study the impact of recombination rates on simulated sequences. To assess the impact of species divergence on the simulated data, we recorded the density of selected SNPs across the whole alignment, as well as the percentage of polymorphic sites in the alignment that were fixed in each population, and we then used *H*_ST_ to estimate differentiation between the two simulated populations.

We used standard ABC procedures to infer appropriate values for parameters of interest [69]. The Euclidean multivariate distance between the observed and simulated normalized summary statistics was calculated, and sets of parameters were accepted and stored if this distance was less than a tolerance threshold, defined so that a small fraction of simulations (less than 1%) were accepted for the estimation step. When comparing the fit of our various models to the data, we used this same rejection threshold and determined the best model using Bayes factors. Posterior distributions of parameters of interest were summarized using average values.

### Intra-specific phylodynamic model

The main genotypes that we observed in *B. burgdorferi* s.s. and *B. garinii*, which were identified by both the phylogenetic and the Structure approaches, likely emerged a long time ago. In order to study how diversity is currently maintained in our sampling sites, we developed an epidemic model that took pathogens diversity into account. More precisely, we explored an individual based model in which the infection status for a number of different bacterial genotypes (*N_bg_*) was monitored over time in hosts and vectors. Our aim was to identify epidemiological settings that would allow for the maintenance of the observed level of diversity within each species.

In this model, one iteration represented one year. Two host populations of equal and constant sizes, *N_hosts_*, were simulated to represent the reservoir of infection-causing bacteria at the Munster and Guebwiller sites. Each host population came into contact with a population of nymphs, with constant and equal populations sizes *N_ticks_*. Due to the complete turnover in nymphs that happens each year, larvae were not explicitly included in the model but implicitly appeared at each generation as a source of new susceptible ticks that are coming in contact with hosts. Likewise, adults were not included, as they occur at lower densities and their preferred host are large mammals, which are not known to be a reservoir of the *B*. *burgdorferi* species complex. At each iteration of the model, each host was exposed to a variable number of nymphs, this number followed a Poisson distribution with parameter *N_ticks_*/*N_hosts_*. The bacterial genotype (or lack thereof) within each tick that fed on each host was recorded to obtain the number of contact between each individual host and each bacterial genotype. A host became infected by the i^th^ bacterial genotype according to a binomial distribution which had as parameters the number of contacts this host had with the i^th^ genotype and the probability *I_THi_* of infection occurring during each contact. Infections were persistent, so that an infected host retained this status for its whole life. Then, the entire tick population was completely replaced with susceptible individuals. These new ticks represented uninfected larvae who, in the current iteration would, feed, become infected, molt into nymphs and then infect hosts during the next iteration. Next, each tick thus fed on a host chosen at random in the population. For each of the *N_bg_* different bacterial genotypes, ticks became infected according to a Bernoulli distribution which took as parameters the probability *I_HTi_* of infection per contact if the host was infected, and 0 if the host was uninfected. Later, *N_mig_* host individuals were exchanged between the two host populations following a Poisson distribution with parameter *F_mig_*/*N_hosts_*, where *F_mig_* is a scaling parameter. Finally, a proportion *R_hosts_* of each host population was renewed with susceptible individuals.

An important criterion in the parameterization of our model was that the number of genotypes present in the model at equilibrium (taking into account genetic drift and migration) was equal the number of observed genotypes in our *B. burgdorferi* s.s. and *B. garinii* samples. Therefore, for each simulation, the model was run for 500 iterations in order to allow enough time to reach this equilibrium. We began each simulation with the assumption that each host, was infected by a single bacterial genotype, and that bacterial genotypes occurred at equal frequencies within and among populations. In such an epidemiological model, one of the two main outcomes is expected: either more pathogens are lost during population replacement than are created by new infections, which leads to the extinction of pathogens; or the number of new infections per iteration is much higher than the number of individuals lost during replacement, which leads to high pathogen prevalence and the maintenance of most genotypes in co-infected individuals. Here, we focused on sets of parameters that would enable the creation of intermediate situations. In order to identify these situations, we started simulations with a high number of genotypes, i.e. *N_bg_* = 20; assumed strong rates of transmission during contact between hosts and ticks, *I_THi_* = *I_HTi_* = 0.8; and, as high *Nticks*/*Nhosts* ratios lead to explosive pathogen dynamics, we assumed that *N_ticks_*/*N_hosts_* = 1. To explore the remaining parameter space, we performed simulations using combinations of the following values: *N_hosts_* set at either 100, 300, 500, or 900 individuals; *F_mig_* set at either 0, 0.2, 0.4, 0.6, 0.8, or 1; and *R_hosts_* set at values from 0.32 to 0.42 with a 0.005 step from one to the next. Fifty simulations were performed for each combination of parameters.

To evaluate the fit of the outcome of each simulation to our observed data, we compared different summary statistics using techniques similar to an ABC approach. At the end of each simulation, a certain number of ticks were randomly selected, with the goal of mimicking our real-world sampling of chromosomal sequences; the number of ticks selected corresponded to our actual sampling effort in each site. For *B. burgdorferi* s.s., 14 pathogens were sampled from 14 infected ticks from the first population (Munster) and 9 pathogens were sampled from the second population (Guebwiller). For *B. garinii*, 13 pathogens were sampled in the Munster population and 19 in the Guebwiller population. We computed the Euclidean multivariate distance between the observed and simulated values using the following normalized summary statistics: i) genotype richness among pathogens sampled in each population (*R_1_*, *R_2_*) and in the two populations combined (*R_T_*); ii) Nei diversity indices within each population (*H_1_*, *H_2_*) and differentiation between populations measured with *H*_ST_. Sets of parameters were accepted and stored if the distance was lower than a tolerance threshold, defined so that less than 1% of simulations were accepted. For *B. burgdorferi* s.s., the observed statistics were *R_1_* = 3, *R_2_* = 2, *R_T_* = 3, *H_1_* = 0.62, *H_2_* = 0.44, and *H*_ST_ = 0.09. For *B. garinii*, observed statistics were *R_1_* = 6, *R_2_* = 6, *R_T_* = 8, *H_1_* = 0.80, *H_2_* = 0.78, and *H*_ST_ = 0.05.

## Results

### Genomic data

We obtained 2,351,440 sequence reads in total, which corresponded to chromosomal coverage ranging from 2.2X to 27.2X, with an average of 8.5X. Raw reads were deposited to the Sequence Read Archive (SRA) database under accession SRP031890. Whole Genome Shotgun projects have been deposited in GenBank under the accessions listed in Table S1 (BioProject PRJNA223339).

The raw sequences obtained for each genome were mapped onto reference sequences of the chromosome and plasmids cp26 and lp54. For the chromosomal sequences, we obtained at least 90% coverage of at least one of the reference sequence for 59 out of the 63 genotypes. The four strains for which we did not find 90% coverage were IPT27 and IPT70 of *B. burgdorferi* s.s. and IPT95 and IPT107 of *B. garinii*, which were subsequently excluded from further chromosomal analyses. For the plasmids cp26 and lp54, we obtained 90% reference sequence coverage for 61 strains, excluding IPT95 (which was also excluded from chromosomal analyses). Strains IPT107 and IPT136 of *B. garinii* did not reach the coverage threshold for cp26 and lp54, respectively, and were therefore excluded from further analyses of those respective plasmids.

To perform SNPs-based analyses, we defined unique sets of markers based on sequence alignments. For the entire species complex, 39757, 1569, and 3658 SNPs were identified along the chromosomal, cp26, and lp54 sequences, respectively. Within species we identified i) 5342, 480, and 403 SNPs in *B. burgdorferi* s.s.; ii) 10119, 451, and 873 in *B. garinii*; and iii) 8498, 568, and 762 in *B. afzelii* (number of sites from chromosomal, cp26, and lp54 alignments, respectively).

### Inter-specific phylogenomic patterns of relatedness

**Figure 2:**
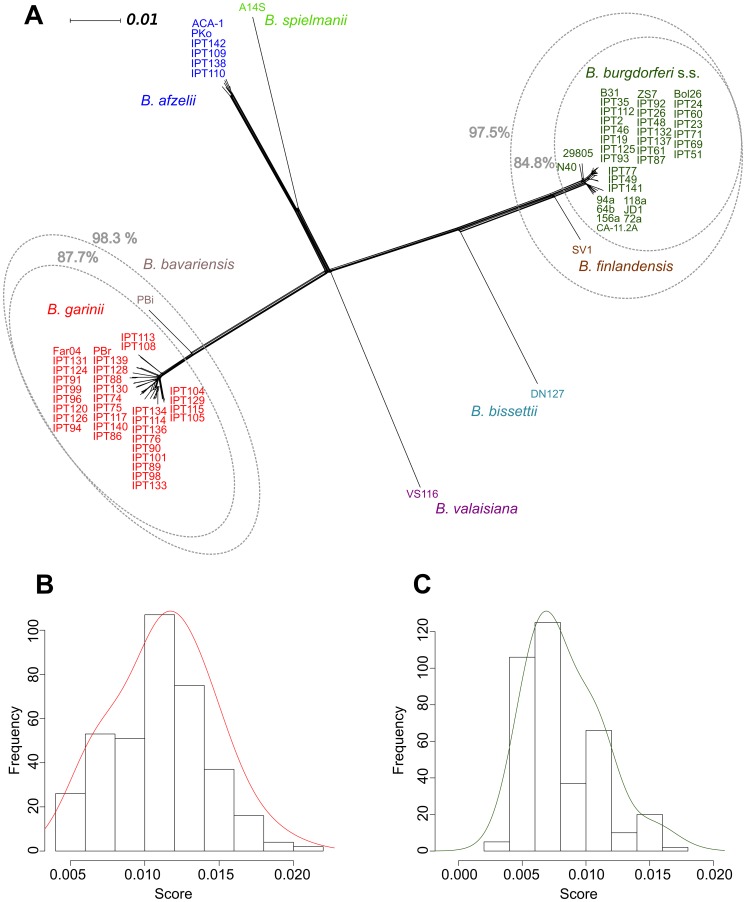
Phylogenetic delineation of species in the *B. burgdorferi* complex based on chromosomal sequences. (A) Neighbor-Net network based on chromosomal sequence data that illustrates phylogenetic relationships among sampled and reference strains of *Borrelia.* The network was constructed with SplitsTree 4 software using the Neighbor-Net method and based on a distance matrix calculated in Paup* 4.0 b10 using a GTR+I+G model. In grey, the percentage of the 1-kb-long contiguous windows obtained from different genetic groups of *Borrelia* that support their monophyly. Distributions of expected probabilities of monophyly for strains of (B) *B. garinii* and (C) *B. burgdorferi* s.s. in the absence of recombination, based on 300 simulations of 1,000,000 genome alignments delineated into 1,000 bp windows. Continuous distributions (in red and green) were obtained using Gaussian kernels.

To illustrate global phylogenetic relationships among the sampled and reference strains at the species level, we constructed a Neighbor-Net network based on chromosomal data (Figure 2). Within this network, the taxonomic assignments of the isolates were consistent with those obtained from PCR-RFLP data (data not shown) and published species boundaries. Within this network, all *B. burgdorferi* s.s. strains included in the network form a clade that is separate from that of strain SV1, for which a unique species name, *B. finlandensis*, has been proposed [70]. Similarly, all the *B. garinii* strains included in the network were more closely related to each other than to the *B. bavariensis* PBi reference strain. However, as previously described [35], the average amount of sequence divergence was low both between *B. burgdorferi* s.s. and *B. finlandensis,* and between *B. garinii* and *B. bavariensis*, species pairs for which we measured respective average divergences around 2.2% and 2.8%.

Contiguous 1-kb windows were obtained from alignments in order to construct region-specific phylogenies and assess the monophyly of *Borrelia* species and/or broader genetic groups using different sets of genetic data (Figure 2, Table 1). Overall, the relationships within these phylogenies were consistent with those observed in the global network, and a high percentage of the windows from each replication unit supported the monophyly of the various *Borrelia* species, although this percentage was slightly lower for the cp26 plasmid (Table 1). In the case of chromosomal data, 85% of the window-based phylogenies supported a clade that contained all the *B. burgdorferi* s.s. strains. Furthermore, 97% of trees supported a larger monophyletic group that included all *B. burgdorferi* s.s. strains as well as the *B. finlandensis* SV1 strain. Similarly, 88% of phylogenies contained a monophyletic group of *B. garinii* strains and nearly 98% of trees supported a clade that included all *B. garinii* strains and the *B. bavariensis* strain PBi. Finally, 99% of phylogenetic trees supported the monophyly of the *B. afzelii* strains.

**Table 1:** Percentage of the 1-kb-long contiguous windows obtained from different genetic groups of *Borrelia* that support their monophyly.

Genetic groups	Chromosome	cp26	lp54
*B. burgdorferi* s.s.	84.8	60.0	83.0
*B. burgdorferi* s.s. and *B. finlandensis* SV1	97.5	86.7	88.7
*B. garinii*	87.7	83.3	83.0
*B. garinii* and *B. bavariensis* PBi	98.3	90.0	96.2
*B. afzelii*	98.7	93.3	98.1
*B. afzelii* and *B. spielmanii* A14S	90.2	83.3	90.6

We also wanted to investigate whether the percentage of windows that did not support the monophyly of *B. burgdorferi* s.s. (15%) and *B. garinii* (12%) could simply be explained by a lack of phylogenetic information, and not the presence of recombination. However, when we generated 1-kb windows from simulated data that did not allow for recombination, the percentages of windows that did not support the monophyly of the two bacterial species were significantly lower than those observed from the actual molecular data (Figure 2).

### Genomic structure among species

*H*_ST_ values, i.e. measures of genetic differentiation based on SNPs, were calculated for each pair of species within the *B. burgdorferi* species complex with the aim of obtaining a quantitative measurement of the degree of genetic isolation among the sampled species (Table 2). In general, we found more differentiation between species in the chromosome and lp54 data than in the cp26 data. When we used SNPs from all replication units to examine the differentiation between *Borrelia burgdorferi s.s.* and each of the two other *B. garinii* and *B. afzelii* species, we found that over 90% of SNPs showed significant differentiation (p<0.05). Likewise, more than 80% of SNP sites were significantly differentiated between sequences of *B. garinii* and *B. afzelii* (data from all three replication units).

**Table 2:** Mean differentiation (*H*_ST_ values) of SNPs identified in different groups of *Borrelia* and the percentage of *H*_ST_ values that demonstrated a significant degree of differentiation (p<0.05).

	Chromosome			cp26			lp54		
Groups compared	Mean	std^a^	S^b^	Mean	std^a^	S^b^	Mean	std^a^	S^b^
*B. burgdorferi* s.s. vs. *B. afzelii*	0.92	1.0e-03	95.2	0.82	7.5e-03	89.2	0.96	2.1e-03	98.0
*B. burgdorferi* s.s. vs. *B. garinii*	0.88	1.2e-03	99.7	0.83	7.5e-03	98.7	0.92	3.2e-03	99.9
*B. garinii* vs. *B. afzelii*	0.79	1.8e-03	85.0	0.73	1e-02	80.2	0.84	4.7e-03	90.3
*B. burgdorferi* s.s., Munster vs. Guebwiller	0.06	6.2e-04	0.52	0.05	2.6e-03	3.29	0.06	3.5e-03	4.6
*B. garinii,* Munster vs. Guebwiller	-0.001	4.3e-04	3.58	0.00	2.2e-03	3.85	-0.005	1.3e-03	2.6

a standard error of the mean.

b percentage of *H*_ST_ values indicating significant differentiation (%).

**Figure 3:**
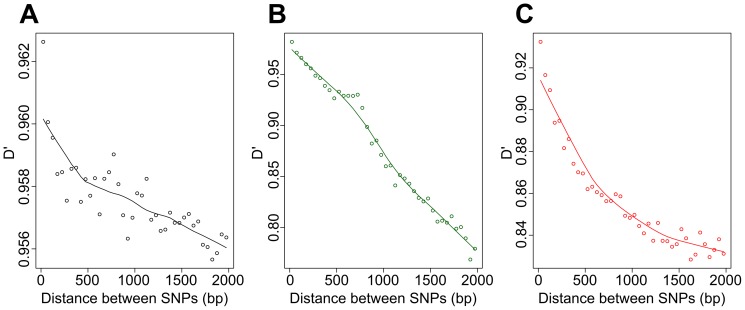
Relationships between D' and the physical distance between SNPs. D' values were calculated from pairs of SNPs using the chromosomal sequences of (A) all strains used in this study, (B) all strains of *B. burgdorferi* s.s., and (C) all strains of *B. garinii*. Average D' values obtained for all pairs of SNPs separated by less than 2,000 bp (using 50-bp intervals) are shown.

After observing this differentiation pattern, we investigated the impact of homologous recombination on the genetic diversity of the *B. burgdorferi* species complex by calculating D′ linkage disequilibrium values for all pairs of SNPs, both in i) all genomes grouped together and ii) within groups of conspecific genomes, assigned to *B. burgdorferi* s.s., *B. garinii,* or *B. afzelii* (Table 3). As expected from our analysis of differentiation, the average D′ value over the three replication units calculated for the set of all strains was high (0.92; standard error of 6.2e-06); it was higher than the values obtained for *B. burgdorferi* s.s. and *B. garinii* (0.75 and 0.81; standard errors of 7.8e-05 and 4.3e-05, respectively) but lower than that of *B. afzelii* (0.96; standard error of 2.9e-05). Nevertheless, this last value should be treated with caution due to the low sample size used to generate it. When we examined the individual replication units in the whole sample, we found a negative relationship between linkage disequilibrium and physical distance, but the decrease of D' values leveled off after 500 bp (e.g. for chromosomal data Figure 3).

**Table 3:** Mean linkage disequilibrium (D' values) at different sampling scale for pairs of SNPs within different genetic groups of *Borrelia.*

	Chromosome		cp26		lp54	
Sampling scales	Mean	std^a^	Mean	std^a^	Mean	std^a^
All strains	0.92	6.2e-06	0.87	2.1e-04	0.94	5.3e-05
All strains from this study	0.96	5.3e-06	0.92	1.8-04	0.98	3.5e-05
All *B. burgdorferi* s.s. strains	0.75	7.9e-05	0.76	9.2e-04	0.66	1.2e-03
*B. burgdorferi s.s.* strains from this study	0.97	4.3e-05	0.95	5.9e-04	0.93	1.1e-03
All *B. garinii* strains	0.81	4.3e-05	0.80	9.8e-04	0.73	5.5e-04
*B. garinii* strains from this study	0.82	4.2e-05	0.81	9.9e-04	0.74	5.5e-04
All *B. afzelii* strains	0.96	2.9e-05	0.92	6.1e-04	0.95	4.6e-04
*B. afzelii* strains from this study	0.98	2.9e-05	0.97	4.7e-04	0.97	2.8e-04

### Intra-specific patterns of relatedness

**Figure 4:**
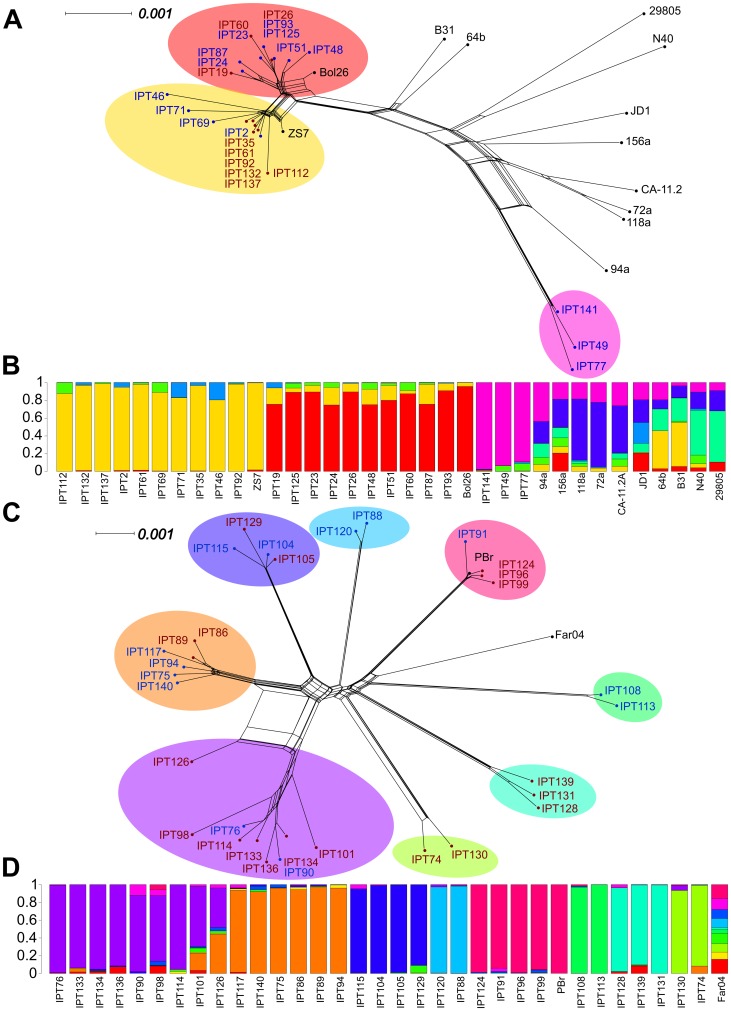
Phylogenies and population structures of *B. burgdorferi* s.s. and *B. garinii*. Neighbor-Net networks based on chromosomal data reveal the phylogenetic relationships among sampled and reference strains of (A) *B. burgdorferi* s.s. and (C) *B. garinii.* Networks were constructed with SplitsTree 4 software and were based on a distance matrix calculated in Paup* 4.0 b10 using a GTR+I+G model. Strain names are surrounded in function of the main population they were assigned to using Structure; the results of the best run of Structure v2.3.4 are shown for (B) *B. burgdorferi* s.s. (K = 7) and (D) *B. garinii* (K = 13). Analyses were based on all polymorphic sites present in at least 90% of strains identified using multiple alignments of chromosomal data, assuming correlations among linked loci and allowing admixture among potential populations.

To more precisely illustrate the genetic relationships among the sampled and reference strains within species, we constructed Neighbor-Net phylogenetic networks based on the chromosomal data of *B. burgdorferi* s.s. and *B. garinii* (Figure 4).

Within *B. burgdorferi* s.s., most strains clustered together to form two closely related phylogenetic groups that included the published sequences of strains Bol26 and ZS7 (Figure 4). This branching pattern was unexpected, as these two published strains had been isolated in Italy and Germany, respectively, with the latter originating from a study conducted more than twenty years ago [71]. Conversely, strains IPT49, IPT77, and IPT141, which were isolated from ticks sampled from a single location in both 2003 and 2004, grouped together in a separate clade and appeared to be more closely related to North American strains of *B. burgdorferi* s.s., such as strain 94A. In fact, most of the reticulations in this region of the network involve branches leading to American strains.

Strains assigned to *B. garinii* formed nine groups of closely related genotypes (Figure 4). These genetic groups were connected in a star-like phylogeny, with the exception of strain IPT126, which was ambiguously connected via reticulations to two different groups of strains (one including IPT75, IPT86, IPT89, IPT94, IPT117, and IPT140, and the other containing IPT76, IPT90, IPT98, IPT101, IPT114, IPT133, IPT134, and IPT136. None of the *B. garinii* strains that were sequenced for this study clustered with the reference strain *B. garinii* Far04, but we found that four of our sampled strains (IPT91, IPT96, IPT99, and IPT124) grouped with the reference strain *B. garinii* PBr, which had been isolated in Germany in the 1980's.

Finally, the network showed that the *B. afzelii* strains isolated in this study were closely related to reference strains PKo and ACA-1. However, it also showed that this group of sampled strains is not monophyletic.

### Population structure within sampled species

We performed Structure analyses to describe the population structure of *B. burgdorferi* s.s. and *B. garinii* in more detail. The population structure of strains of *B. burgdorferi* s.s. suggested an optimal value of K = 7 populations (Figure S1). Results revealed that European strains were assigned to three main populations, which showed low levels of admixture (Figure 4). Conversely, high levels of admixture were identified among American strains. For *B. garinii*, the optimal number of populations was computed to be K = 13 populations, and strains from this study were assigned to 9 of those 13 populations, with strain IPT126 showing a high degree of admixture (Figure 4).

We then compared in more detail the distribution of genetic diversity within the three sampled species by calculating two estimates of the diversity statistic θ (measured per site): θ_S_ and θ_π_. From the chromosomal data of each species, overall estimates confirmed that strains of *B. garinii* contained more genetic diversity than those of *B. burgdorferi* s.s. or *B. afzelii* (Table 4). Among our samples, in *B. burgdorferi* s.s. we found that θ_S_ = 2.7e-03 and θ_π_ = 2.0e-3. For *B. garinii*, θ_S_ and θ_π_ were 6.3e-03 and 5.5e-03, respectively and in the case of *B. afzelii*, θ_S_ was 4.5e-03 and θ_π_ was 4.6e-03.

**Table 4:** Chromosomal genetic diversity and Tajima's D values.

	θ_S_		θ_π_		Tajima's D	
Species	Mean	std^a^	Mean	std^a^	Mean	std^a^
All *B. burgdorferi* s.s. strains	4.2e-03	9.4e-05	3.1e-03	1.6e-04	-1.02	4.1e-02
*B. burgdorferi* s.s. strains from this study	2.7e-03	7.2e-05	2.0e-03	9.3e-05	-0.80	5.9e-02
All *B. garinii* strains	6.6e-03	8.7e-05	5.5e-03	8.7e-05	-0.58	2.1e-02
*B. garinii* strains from this study	6.3e-03	8.6e-05	5.5e-03	8.9e-05	-0.44	2.1e-02
All *B. afzelii* strains	4.0e-03	1.1e-04	3.6e-03	1.2e-04	-0.33	3.9e-02
*B. afzelii* strains from this study	4.4e-03	1.3e-04	4.5e-03	1.4e-04	0.18	7.1e-02

a standard error of the mean.

To estimate the diversity statistic θ, Watterson's θ_S_ and Tajima's θ_π_ were calculated using chromosomal sequence data for all 1-kb windows. Tajima's D values were calculated from these estimates; the mean and standard error for each group are shown.

**Figure 5:**
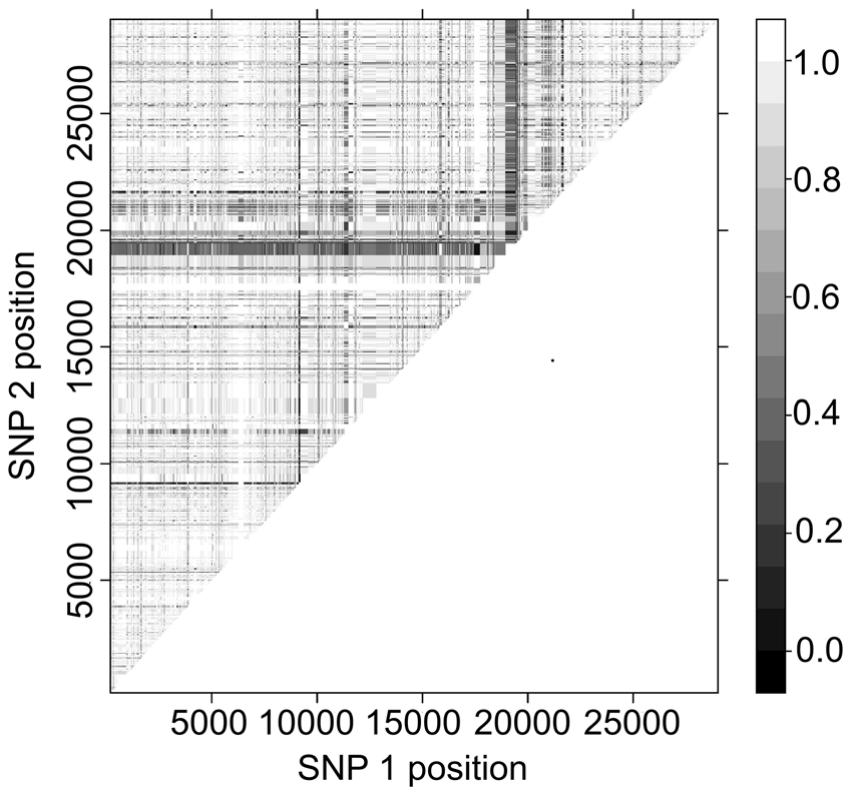
Linkage disequilibrium along the cp26 plasmid. The axes are the positions on the plasmid of the SNPs included in each pairwise analysis. An absence of shading corresponds to a high level of linkage disequilibrium (D' close to 1) and progressively darker shading indicates decreasing values of D'. The genome region in and around the *ospC* gene is characterized by low linkage disequilibrium.

Next, we focused on pairwise D′ values for SNPs within either the *B. burgdorferi* s.s. strains or the *B. garinii* strains from both of our sampling sites. Interestingly, these values were higher and, in the case of *B. burgdorferi* s.s., considerably higher than those that we calculated from all available genomes (Table 3). For the three replication units taken together, the average D' value was 0.97 for *B. burgdorferi* s.s. and 0.81 for *B. garinii* (respective standard errors of 4.3e-05 and 4.2e-05). As for inter-specific data, we found a negative relationship between linkage disequilibrium and physical distance within species, and the decrease of D' values especially leveled off after 500 bp in *B. garinii* (Figure 3). Within both species, we found SNPs with very low D' values, indicating low levels of linkage disequilibrium with most other SNPs and a large number of these SNPs were located in the vicinity of the *ospC* gene (Figure 5).

**Figure 6:**
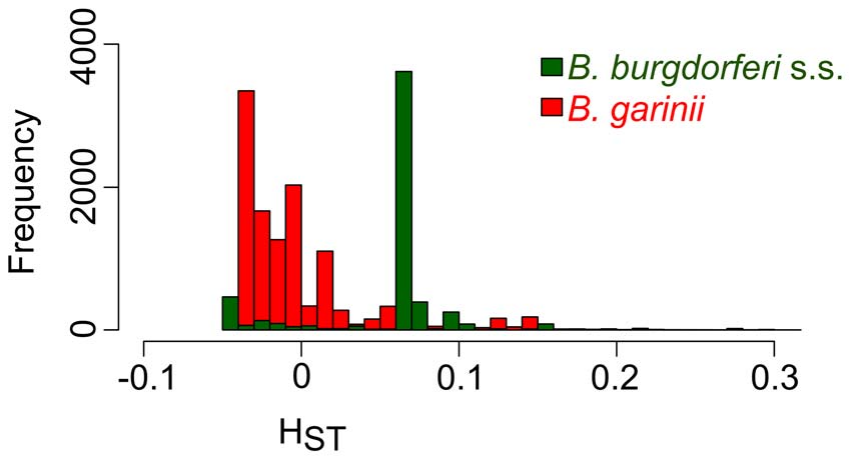
Genetic differentiation between sampling sites for *B. burgdorferi* s.s. and *B. garinii*. Distributions of H_ST_ values were obtained from within-species analyses of geographical genetic differentiation based on SNPs. Genetic differentiation between the two sites was significantly higher for *B. burgdorferi* s.s. than for *B. garinii*.

Given that the levels of linkage disequilibrium in *B. burgdorferi* s.s. and *B. garinii* in our regional sample were higher than those calculated for the global sample, we investigated the extent of geographical isolation within each species by using SNPs frequencies to analyze the distribution of *H*_ST_ values between our two sampling sites (Table 2, Figure 6). At the SNP level, only a few markers (ranging from 0.52% to 4.58% of SNPs per species/replication unit combination) revealed significant isolation (p<0.05) between samples obtained from Munster and those obtained from Guebwiller for either *B. burgdorferi* s.s. or *B. garinii*. However, the distribution of *H*_ST_ values obtained from isolates of *B. burgdorferi* s.s was centered around a significantly higher value (p<2.2e-16) than the distribution of values obtained from *B. garinii*, indicating that the former species was significantly more differentiated between our two study sites (Figure 6). In both species, *H*_ST_ values computed from SNPs located in a region of 4 kb around the *ospC* gene on cp26 deviated strongly from the rest of the distribution (Figure S2), a signal that this region is subjected to peculiar evolutionary constraints. Similarly atypical patterns could be observed in various regions of the chromosome and of the lp54 plasmid (Figure S2).

### Evidence of diversifying selection along replication units

Tajima's D values were computed for 1-kb windows of sequence data obtained from each replication unit for each of the sampled species. Within each individual species, most of the mean Tajima's D values for the individual replication units were negative (Figure S3): for *B. burgdorferi* s.s., mean Tajima's D values were −1.02, −0.13, and −0.61 for the chromosome, cp26 and lp54 plasmid, respectively; for *B. garinii*, they were −0.58, −0.46, and −0.58, respectively; and for *B. afzelii* they were −0.33, 0.12, −0.37 respectively.

Because strong Tajima's D values could be the result of host-driven selective pressures on the genes contained within the windows and indicate genome regions showing extensive genetic diversity, we identified the 1-kb windows within each species that had the highest absolute values. When we identified the genes that were present within the selected windows, we found that, compared to the total number of annotated genes for each of the three replication units (chromosome, cp26, and lp54), the genes within these highlighted windows contained a significantly lower proportion of cytoplasmic protein-encoding genes and, instead, were significantly enriched in surface lipoprotein-encoding genes (Fisher's exact test, p<0.05). More precisely, genes that encoded cytoplasmic proteins (identified through SLEP analyses) only represented 55% of genes with high Tajima's D values even though they made up 67% of the genes within the total proteome encoded by the chromosome, cp26, and lp54. Genes annotated as encoding for membrane-associated proteins made up 23% of genes with high Tajima's D values and 22% of genes in the studied proteome, while genes described as encoding exported proteins represented 4% and 5% of those respective groups. Finally, 18% of the genes with high Tajima's D values were annotated as lipoprotein-encoding genes, which make up only 6% of genes in the proteome as a whole. Another observation adds weight to the unique position of lipoprotein-encoding genes in this sample is that the only gene found in high-D-value windows in all three *Borrelia* species was the *ospC* lipoprotein gene.

### Phylogenetic analysis of the *ospC* gene and flanking regions

**Figure 7:**
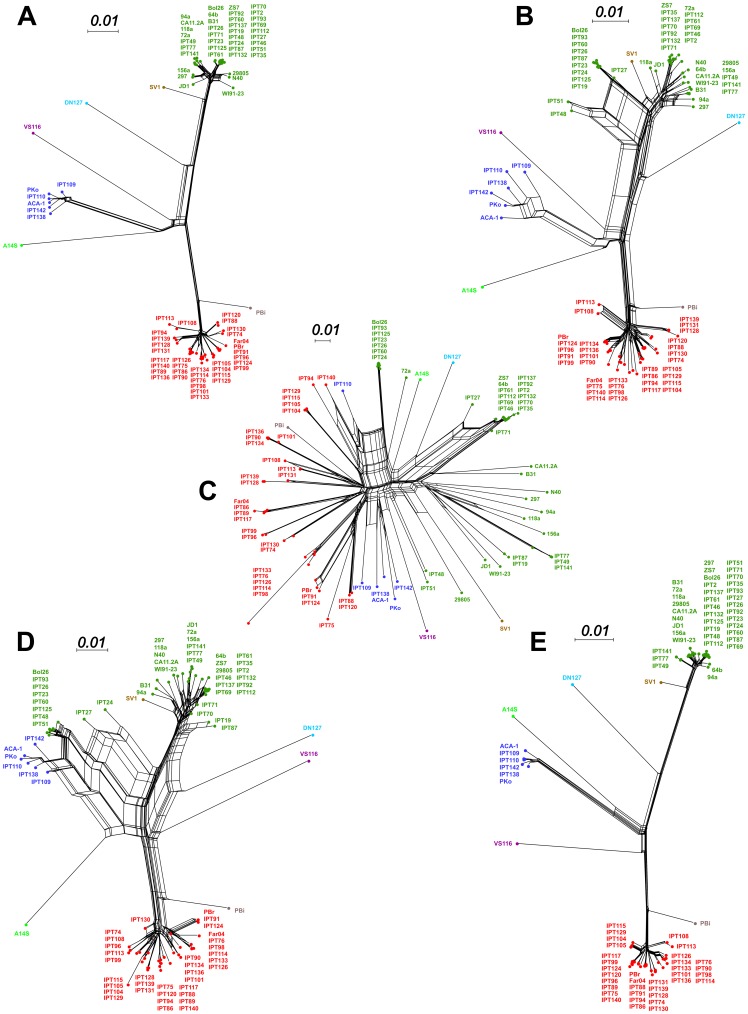
Phylogenetic ambiguities in the ospC gene in lineages associated with small mammals. Five Neighbor-Net networks were obtained (using p-distances and SplitsTree 4) from alignments of the regions including and flanking the *ospC* gene and are presented in the order in which they physically occur in the cp26 sequence. (A) Upstream region; (B) 2,000 bp sequence that occurs before *ospC*; (C) the *ospC* gene, (D) 2,000 bp sequence that occurs after *ospC*; and (E) downstream region delineated.

In order to more thoroughly investigate the reasons for which the region around the *ospC* gene produced peculiar results in our analyses of linkage disequilibrium, genetic differentiation, and Tajima's D values, we constructed phylogenetic networks of the *ospC* gene and flanking regions (Figure 7). The network obtained from the alignment of *ospC* sequences showed a star-like structure, with most external branches including a small number of genotypes (Figure 7C). When we examined either the 2,000 bp upstream or the 2,000 bp downstream of the *ospC* gene, the structure became more resolved (Figures 7B and 7D). Nevertheless, all of these networks differed markedly from the one based on the chromosomal alignment (Figure 2). Most notably, in these networks using cp26 data, *B. burgdorferi* s.s. did not form an obvious clade. Its constituent strains were distributed into different subgroups that were linked by large reticulations to strains of either *B. afzelii* or *B. spielmanii.* Furthermore, *B. finlandensis* strain SV1 was included within a group of *B. burgdorferi* s.s. strains. Conversely, *B. burgdorferi* s.s. strains IPT49, IPT77, and IPT141, for which the phylogenetic analysis based on chromosomal data had revealed only a distant relationship to other *B. burgdorferi* strains, were still grouped together but were embedded among other *B. burgdorferi* s.s. strains. When we constructed networks based on sequence data further upstream or further downstream of the *ospC* gene, the patterns became more consistent with the phylogenetic signal obtained from the chromosome (Figure 7A and 7E).

### Long-term coalescent based model

We developed coalescent-based models to estimate the impact of various divergence times, effective population sizes, and intra-specific and inter-specific recombination rates on the evolutionary history of *B. burgdorferi* s.s. and *B. garinii*. First, we checked that the distributions of summary statistics obtained from our accepted simulations contained the statistics generated from our observed data (data not shown). To obtain quantitative support for our inference, we used Bayes factors to compare different models that had different constraints applied to the parameters of interest (Table S4). As a result of this comparison, we chose the M3 model for further analysis. From posterior distributions of the parameters of interest (Figure S4), we computed average values for each parameter and scaled the different estimates to yield interpretable results. The resulting estimates of the θ parameter were 8.2e-04 for *B. burgdorferi* s.s. and 1.5e-03 for *B. garinii*, while the effective population sizes of the two species (assuming a mutation rate of 1e-07 per site per generation) were approximately 8200 and 14500 respectively. The inter-specific recombination rates, *r_inter1_* = *r_inter2_*, was estimated to be 3.1e-09, which was 50 times lower than the intra-specific recombination rates, *r_intra1_* = *r_intra2_* = *r_intra3_* = 1.7e-07. The ratio of the recombination rate to the mutation rate was approximately 1.7. The model estimated that the two species shared a common ancestor around 490,000 generations ago. The ancestral population size estimate should be treated with caution as the posterior distribution from which it came indicates a large degree of uncertainty regarding the outcomes of the model (Figure S4).

### Intra-specific phylodynamic model

**Figure 8:**
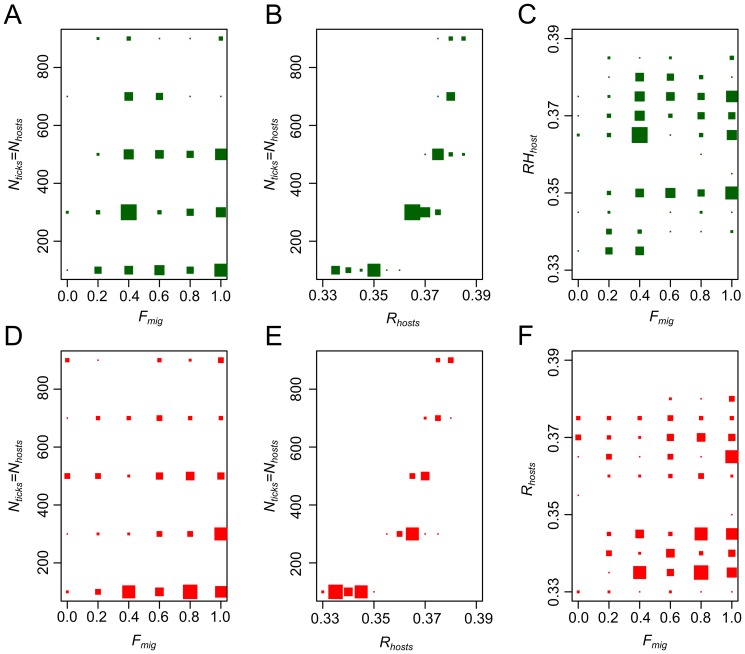
Distribution of the parameter values of the phylodynamic model simulations. The values of the parameters of interest for the simulations we selected (based on their similarity to our observations) are plotted. Green points correspond to simulations of *B. burgdorferi* s.s. and red points to simulations of *B. garinii* and: (A and D) *F_mig_* in function of *N_ticks_ = N_hosts_*; (B and E) *R_hosts_* in function of *N_ticks_ = N_hosts_*; (C and F) *F_mig_* in function of *R_hosts_*.

We explored the properties of an individual-based epidemiological model to better understand factors that could influence the maintenance of the diversity we observed in our two sites, Munster and Guebwiller. Independently for each species, we performed 50 simulations that assumed different values for parameters of interest: the number of hosts per population, *N_hosts_*; the replacement rate of the host population, *R_hosts_*; and the migration rate between sampling sites *F_mig_/N_hosts_*. Of these simulations, we selected those whose summary statistics were similar to observed values (1% of the total simulations for *B. burgdorferi* s.s. and *B. garinii*). We then plotted the values of the parameters for the selected simulations in order to identify patterns of epidemiological relevance (Figure 8). Overall, larger population sizes and higher migration rates were required to maintain the diversity that we observed in *B. garinii* than were necessary for the maintenance of observed patterns in *B. burgdorferi* s.s. According to the model, host population sizes of l00 individuals would be sufficient to function as effective reservoirs for the diversity we observed in our study.

## Discussion

### Distribution of diversity among species of the *Borrelia burgdorferi* complex

We studied species delineations within the *B. burgdorferi* complex using genomic data from sympatric isolates and geographically independent reference sequences. Our results show that the *B. burgdorferi* species complex is composed of different genetic groups that are clearly isolated from one another (Figure 2). These results are consistent with the robust genetic boundaries that have been observed among the members of the *B. burgdorferi* species complex following analyses of concatenated MLST data [29]. Although there were few obvious inter-specific recombination events, most phylogenies based on contiguous 1-kb windows along the three main replication units studied here (the chromosome, the cp26 plasmid, and the lp54 plasmid) showed clear delineations among the *B. burgdorferi* s.s., *B. garinii*, and *B. afzelii* strains (Table 1). This finding is further supported by the almost complete genetic differentiation that we observed in our analysis of *H*_ST_ values among the three species (Table 2). Finally, linkage disequilibrium measures were much higher for the species complex as a whole than within the two most frequently sampled species, *B. burgdorferi* s.s. and *B. garinii* (Table 3). Additionally, coalescent-based inference consistently revealed that within-species recombination rates for *B. burgdorferi* s.s. and *B. garinii* may be more than ∼50 times higher than between-species recombination rate (Figure S4). Taken together, these data suggest that homologous recombination occurs much more frequently within than among species, which counters previous suggestions that, the rate of cross-species recombination in this species complex is high [36].

Some exceptions to the monophyly of the sampled species were found: *B. burgdorferi* s.s. was non-monophyletic in 15% of the phylogenies generated here, and *B. garinii* was non-monophyletic in 12% (Figure 2). The majority of these incongruencies resulted from the inclusion of the *B. finlandensis* SV1 strain within a cluster of *B. burgdorferi* s.s. strains or the inclusion of the *B. bavariensis* PBi strain with strains of *B. garinii*. However, the average amount of sequence divergence in both cases (between *B. burgdorferi* s.s. and *B. finlandensis*, and between *B. garinii* and *B. bavariensis*) is lower than the 5% divergence usually observed among *bona fide* species [72]. This raises questions on the amount of information available to delineate these groups. Additionally, the observed percentages of windows that did not support the respective monophyly of our two study species were much higher than would be predicted by an evolutionary model lacking recombination, as our phylogenetic simulations revealed (Figure 2). Thus, recombination presents itself as a possible phenomenon that might be responsible for the conflicting phylogenetic signal among windows. However, another explanation is also likely: the inability of certain phylogenies to distinguish between and to separate these pairs of species might simply result from the incomplete sorting of closely related bacterial lineages since the time of divergence [73]. This latter hypothesis is supported by the low ratio between the inter-specific and the intra-specific rate of recombination that was observed in *B. burgdorferi* s.s. and *B. garinii*.

This sharp decrease between inter-specific and the intra-specific recombination rates in the *B. burgdorferi* species complex, shown by both the observed genomic data and the results of our coalescent-based model, could be explained by various hypotheses. From an ecological point of view, a lack of physical contact between genetic groups in the species complex as a result of association with different vertebrate hosts may result in divergence if mutations accumulate via genetic drift and/or selection [29], and such physical isolation may prevent the homogenization of lineages via homologous recombination. The divergence of *B. garinii* from *B. bavariensis* could be explained by this hypothesis, as it is associated with a shift in host range [18]. However, no such evidence is available to explain the separation of *B. finlandensis* from *B. burgdorferi*. We also did not find obvious evidence for homologous recombination between *B. valaisiana* and the studied *B. garinii* genomes, even though both species co-infect the same host species in our sampling location [38]. Thus, it seems that a lack of opportunity is not the only factor inhibiting a higher recombination rate. Other hypotheses could be that inter-specific recombinants are genetically less fit and do not persist in populations, or that genetic divergence among species might depress homologous recombination rates in this species complex. In this context, looking for genes experiencing diversifying selection and presenting a high degree of divergence is particularly interesting.

### Evidence of selective pressures within genomes and resulting *ospC* diversification

We used Tajima's D statistic to determine which genes might be affected by selective pressures, and we found that genes involved in lipoprotein production had a greater representation among genes with high Tajima's D values than would have been predicted from their prevalence in the overall proteome of each of the species studied. In this, our results concur with the genetic evidence reviewed by Brisson *et al*. [34] as well as a recent phylogenetic analysis of the ratio of non-synonymous to synonymous mutation rates [35]. Of the lipoprotein-encoding genes highlighted here, the only one identified in all three taxa was *ospC*. The high genetic diversity of the *ospC* gene is probably influenced by a combination of selective pressures. The first type of selective pressure may be a consequence of the function of the lipoprotein encoded by *ospC*, which binds the tick salivary protein Salp15 [74] as well as host plasminogen [75] in order to facilitate *Borrelia* dissemination within hosts. Disruptive selective pressures on the various alleles of *ospC* could give these bacteria the ability to interact with different host species. Indeed, the multiple niche model has been suggested to be a critical driver of the diversity of these bacteria [32]. Second, the protein encoded by *ospC* has antigenic properties [76] and might thus evolve under negative frequency-dependent selective pressure [77]. Evidence of both of these types of selective pressure on *ospC* diversity was obtained from a recent study [33] that found: i) a strong association between bacterial *ospC* genotypes and the rodent hosts they infect, suggesting their limited spread in the host community; and ii) different *ospC* alleles associated with a common genetic background, suggesting the influence of diversifying selective pressure.

We did not find direct evidence of associations between *ospC* alleles and potential host species. However, our phylogenetic analysis of the flanking regions of *ospC* revealed ambiguous relationships between *B. burgdorferi* s.s. and both *B. afzelii* and *B. spielmanii*, an observation that probably indicates some degree of recombination among these taxa despite high levels of divergence. As recombination requires physical contact between genotypes, the recombinant genotypes we observed provide evidence that co-infections with *B. burgdorferi* s.s., *B. afzelii*, and *B. spielmanii* strains occurred in the past. *B. burgdorferi* s.s. can be found in both birds and small mammals, while the two other species are usually associated with small mammals. Therefore, one explanation might be that the *B. burgdorferi* s.s. lineages we sampled are infected the same hosts as *B. afzelii* and *B. spielmanii*. The reticulations in the *ospC* phylogeny suggest that inter-specific recombination may thus be involved in the emergence of new genotypes within the *B. burgdorferi* species complex (Figure 7). However, the lack of evidence of *ospC* recombination between *B. bavariensis* PBi strain and other species that infect small mammals indicates that other evolutionary scenarios are also likely. Finally, it is difficult to estimate the approximate time periods during which these inferred recombinations may have occurred. If recombinations were more recent, it would suggest that the strains of *B. burgdorferi* s.s. that we sampled frequently infect small mammals within the geographical area we studied. A future challenge for researchers will be to calibrate a molecular clock for the *ospC* region that takes into account its specific evolutionary constraints, i.e. high recombination rate and selective pressures.

When we examined the distribution of *ospC* diversity among lineages, we found closely related genomic backgrounds associated with different *ospC* alleles (Figure 7C); this was observed in both *B. burgdorferi* s.s. and *B. garinii*. We also observed a lower degree of linkage disequilibrium around the *ospC* region than in any other genome location (Figure 5). These observations could be explained by a locally higher recombination rate and/or selective pressure driven by hosts' immune systems. Considering the selective pressures likely acting on the *ospC* region, it seems probably that recombination could produce genetic combinations that would subsequently be maintained in higher numbers by diversifying selection than would persist as a result of neutral evolution or purifying selection. However, in describing multiple *ospC* alleles associated with similar genetic backgrounds and the low linkage disequilibrium between *ospC* data and other parts of the genome, our results differ substantially from those of Haven *et al.* [36] who observed a strong relationship between allelic variation in *ospC* and the rest of the genome. The authors of that study used this relationship to hypothesize a major role for negative frequency-dependent selection in the diversification of the *B. burgdorferi* species complex, a role that our results call into question.

### Genetic structure within *Borrelia burgdorferi* s.s

In addition to the patterns observed at the *ospC* locus, our analyses also conflicted with previous observations regarding linkage disequilibrium. Within *B. burgdorferi* s.s., our random sampling at a regional scale revealed much higher linkage disequilibrium values that what can be obtained from previously sequenced genomes [36], [37] (Table 3). This observation was strengthened by a Structure analysis. While the strains we sampled and sequenced, as well as the two other European genomes included here, were assigned to clearly delineated populations, genomes obtained from North American strains showed marked evidence of admixture (Figure 4B). This difference was also visible in the phylogenetic network of *B. burgdorferi* s.s. strains, in which reticulation mostly involved branches leading to American strains (Figure 4A). Originally, the sequenced American strains were “chosen to cover a large fraction of the genetic and geographic diversity” within *B. burgdorferi* s.s. and such a choice might lead the emergence of peculiar diversity patterns [37]. For example, in an analysis that used these American strains, Haven *et al*. described a positive relationship between linkage disequilibrium and physical distance [36], which was counter-intuitive [34]. However, the analysis of our local sample revealed the expected negative relationship between the two statistics (Figure 3). Additionally, the restriction of high values of linkage disequilibrium to genomic areas distant from less than a kilobase that we observed is consistent with current knowledge on the length of DNA fragment exchanged by homologous recombination [34]. The observed difference in linkage disequilibrium patterns between our regional samples and the whole sample, including Schutzer *et al.* genome sequences [37], may be compatible with the hypothesis of an epidemic population structure in *B. burgdorferi* s.s., even though this hypothesis has been previously criticized [36]. The high degree of linkage disequilibrium measured here is probably due to the prevalence in our sample of a small number of bacterial genotypes that had high transmission success, and an analysis of a collection of genomes that optimizes diversity might show more evidence of genome-wide recombination, a pattern that would be coherent with that given by Maynard Smith *et al.* in their seminal paper [13]. In this context, it would be interesting to determine whether the measured difference in linkage disequilibrium measures is due to either a difference in sample selection based on *a-priori* genomic information, or a difference in the geographic scale of sampling. Indeed it has been described in *B. afzelii* that different recombinant genotypes were isolated from independent locations [78].

### Differences between the genetic structures of *B. garinii* and *B. burgdorferi* s.s. and epidemiological considerations

Linkage disequilibrium values within *Borrelia garinii* genomes sampled in Alsace were lower than those measured within strains of *B. burgdorferi* s.s from the same locations (Table 3). Nevertheless, the Structure analysis did not reveal extensive evidence of admixture, with the exception of strain IPT126 (Figure 4C). Further, coalescent-based modeling suggested that recombination rates were similar in the two species. Assuming the same mutation rate for each species and considering only the strains we sampled for this study, we inferred a within-species ratio of recombination rate to mutation rate of 1.7 in *B. burgdorferi* s.s. and *B. garinii*, figures that were in close agreement with a previous estimation based on three pairwise comparisons of related genomes (using chromosome, cp26 and lp54 data) within *B. burgdorferi* s.s. [36]. We observed clearer differences between *B. burgdorferi* s.s. and *B. garinii* from our analysis of population structure, in which we estimated effective population sizes and analyzed genetic differentiation between Munster and Guebwiller. As expected from our measurements of diversity, our coalescent models suggested that the effective population size of *B. burgdorferi* s.s. was lower than the effective population size of *B. garinii*. Moreover, differentiation measures, which are inversely proportional to migration rate, were higher for *B. burgdorferi* s.s. than for *B. garinii*.

However, these measures, by themselves, may not directly help in understanding the observed diversity patterns. The phylogenetic relationships between our sequences and reference sequences, which were isolated years ago in different areas, suggested that the diversification of the observed lineages of *B. burgdorferi* s.s. and *B. garinii* greatly preceded the establishment of the studied transmission networks. In order to more directly investigate current patterns of diversity, then, we implemented a simple phylodynamic model, which did not examine the diversification phase of the different genotypes but focused only on the maintenance of diversity. This model confirmed that the maintenance of the observed level of diversity requires a higher host population size and higher host migration rates in *B. garinii* than in *B. burgdorferii* s.s. These results are similar to those obtained from MLST data in a comparison of the genetic structures of rodent-associated *B. afzelii* and bird-associated *B. garinii* at a broader geographic scale [30]. The phylodynamic model thus provided this study with two important benefits. From an applied point of view, the phylodynamic information about the genetic structure of *B. burgdorferi* s.s. and *B. garinii* was coherent with the phylogenetic signal identified in the *ospC* gene region and both sets of results support the hypothesis that small mammals might be the reservoir of the *B. burgdorferi* s.s. strains studied here. From a purely academic point of view, a simple phylodynamic model that does not assume hypotheses about selective pressures contrary to multiple-niche model and negative frequency-dependent selection, allows researchers to explain the maintenance of numerous bacterial lineages with realistic host population sizes. We thus advocate that further efforts are required to incorporate explicit epidemiological constraints into evolutionary models in order to study the genome of both tick-borne and host associated strains and obtain further insight into the evolution of the *B. burgdorferi* species complex.

## Supporting Information

Figure S1 **Mean likelihood values of Structure runs.** Structure analyses were conducted using K values ranging from 1 to 15; for each value, the analysis was repeated five times and the mean likelihood value is plotted here. Analyses were based on all the polymorphic sites identified in at least 90% of *B. burgdorferi* and *B. garinii* strains using multiple chromosomal alignments. For each species, analyses were performed assuming correlations among linked loci and allowing admixture among potential populations. Green points correspond to results for *B. burgdorferi* s.s. and red points to results for *B. garinii.*
(TIFF)

Figure S2 **Genetic differentiation between isolates from Munster and Guebwiller for** ***B. burgdorferi*** **s.s. and** ***B. garinii*** **.** The distributions of *H*_ST_ values were obtained from within-species comparisons of SNPs between isolates from Munster and Guebwiller for *B. burgdorferi* s.s. (left column) and *B. garinii* (right column). The sequence alignments used in the comparisons were obtained from (A and B) the chromosome, (C and D) the cp26 plasmid, and (E and F) the lp54 plasmid. Red circles correspond to values that showed a significant level of differentiation (*p*<0.05).
(TIFF)

Figure S3 **Distribution of Tajima's D values obtained from contiguous 1-kb windows for the three species of** ***Borrelia*** **included in this study.** Values were calculated for A) chromosomal, B) cp26, and C) lp54 alignments. Green lines indicate the mean value of Tajima's D. Blue lines represent the mean of Tajima's D values calculated from chromosomal data and are replicated in each plot. Red lines indicate the 5^th^ and 95^th^ percentiles of chromosomal Tajima's D values for each species.
(TIFF)

Figure S4 **Posterior distributions of parameters of the coalescent model M3.** Distributions were obtained from the simulations, of the coalescent model M3, we selected (based on their similarity to our observations); (A) *N_1_* = c_1_*N, the population size of *B. burgdorferi* s.s., (B) *N_2_* = c_2_*N, the population size of *B. garinii*, (C) *N_3_* = c_3_*N the ancestral population size, (D) the recombination rate within the *B. burgdorferi* s.s. species *r_intra1_*, (E) the recombination rate within the *B. garinii* species *r_intra2_*, (F) the recombination rate within the ancestral population *r_intra3_*, (G) the inter-specific recombination rate *r_inter1_*, (H) the inter-specific recombination rate *r_inter2_*, (I) M*(c_1_*N+c_2_*N) the time backward until the two populations merged.
(TIFF)

Table S1 **Description of the 63 strains of the** ***B. burgdorferi*** **species complex that were isolated and sequenced in this study.** ^A^: M: Adult male; F: Adult female; N: Nymph. All strains were isolated from *Ixodes ricinus* ticks.
(DOC)

Table S2 **Percentage of the length of reference sequences onto which raw sequences were mapped for each strain of the** ***B. burgdorferi*** **species complex examined in this study.** Reference sequences came from i) the chromosome (Chr), the circular plasmid cp26, and the linear plasmid lp54 of *B. burgdorferi* s.s. strain B31; ii) the chromosome of *B. bavariensis* strain PBi and the cp26 and lp54 plasmids of *B. garinii* strain Far04; iii) the chromosome, cp26 plasmid, and lp54 plasmid of *B. afzelii* strain PKo.
(DOC)

Table S3 **Description of the 23 sequenced strains isolated and sequenced in previous studies and used as references in this study.**
(DOC)

Table S4 **Bayes factors for each pair of coalescent models.** Each cell gives the ratio of the number of simulations selected for the column model to the number selected for the row model (i.e., column: row).
(DOC)
