# Supplementary figures and images for: Comparative Population Genomics of the Borrelia burgdorferi Species Complex Reveals High Degree of Genetic Isolation among Species and Underscores Benefits and Constraints to Studying Intra-Specific Epidemiological Processes
Source: PLoS One. 2014 Apr 10;9(4):e94384. doi: 10.1371/journal.pone.0094384 (PMC3993988; doi:10.1371/journal.pone.0094384)

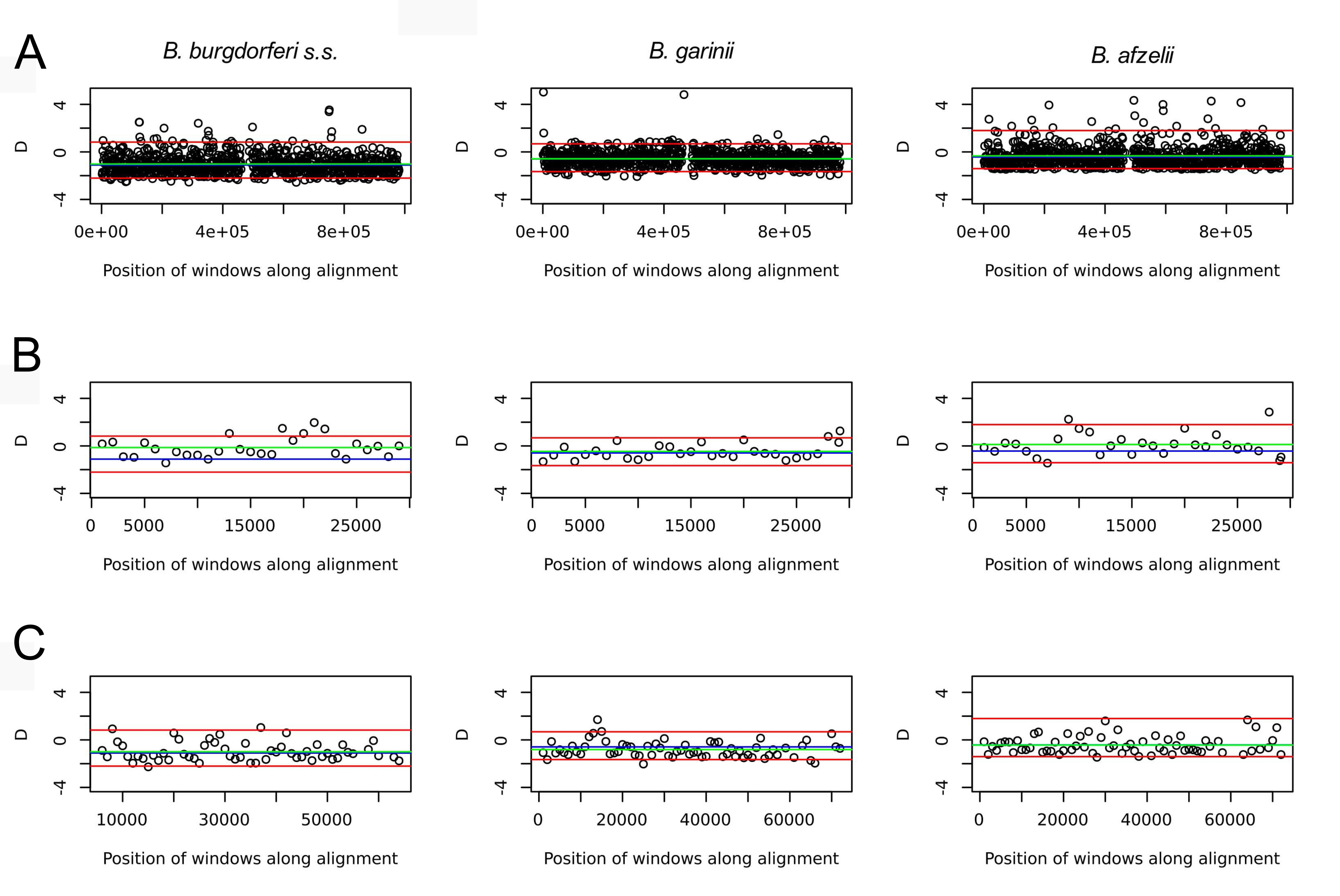

Supplement: Figure S3 — Distribution of Tajima's D values obtained from contiguous 1-kb windows for the three species of Borrelia included in this study. Values were calculated for A) chromosomal, B) cp26, and C) lp54 alignments. Green lines indicate the mean value of Tajima's D. Blue lines represent the mean of Tajima's D values calculated from chromosomal data and are replicated in each plot. Red lines indicate the 5th and 95th percentiles of chromosomal Tajima's D values for each species. (TIFF) [file pone.0094384.s003.tiff]
