# Supplementary material for: Comparative Population Genomics of the Borrelia burgdorferi Species Complex Reveals High Degree of Genetic Isolation among Species and Underscores Benefits and Constraints to Studying Intra-Specific Epidemiological Processes
Source: PLoS One. 2014 Apr 10;9(4):e94384. doi: 10.1371/journal.pone.0094384 (PMC3993988; doi:10.1371/journal.pone.0094384)
Supplement: Table S1 — Description of the 63 strains of the B. burgdorferi species complex that were isolated and sequenced in this study. A: M: Adult male; F: Adult female; N: Nymph. All strains were isolated from Ixodes ricinus ticks. (DOC) [file pone.0094384.s005.doc]

**Table S1. Description of the 63 strains of the *Borrelia burgdorferi* species complex that were isolated and sequenced in this study.**

| **Strain** | Species | Sampling location | Development stage/sex^A^ | Year |
| --- | --- | --- | --- | --- |
| IPT74 | *B. garinii* | Guebwiller | F | 2004 |
| IPT75 | *B. garinii* | Munster | M | 2004 |
| IPT76 | *B. garinii* | Munster | M | 2004 |
| IPT86 | *B. garinii* | Guebwiller | M | 2004 |
| IPT88 | *B. garinii* | Munster | M | 2004 |
| IPT89 | *B. garinii* | Guebwiller | F | 2004 |
| IPT90 | *B. garinii* | Munster | M | 2004 |
| IPT91 | *B. garinii* | Munster | M | 2004 |
| IPT94 | *B. garinii* | Munster | M | 2004 |
| IPT95 | *B. garinii* | Munster | F | 2004 |
| IPT96 | *B. garinii* | Guebwiller | F | 2004 |
| IPT98 | *B. garinii* | Guebwiller | F | 2004 |
| IPT99 | *B. garinii* | Guebwiller | F | 2004 |
| IPT101 | *B. garinii* | Guebwiller | F | 2004 |
| IPT104 | *B. garinii* | Munster | F | 2004 |
| IPT105 | *B. garinii* | Guebwiller | F | 2004 |
| IPT107 | *B. garinii* | Munster | M | 2004 |
| IPT108 | *B. garinii* | Munster | M | 2004 |
| IPT113 | *B. garinii* | Munster | F | 2004 |
| IPT114 | *B. garinii* | Guebwiller | M | 2004 |
| IPT115 | *B. garinii* | Munster | F | 2004 |
| IPT117 | *B. garinii* | Munster | F | 2004 |
| IPT120 | *B. garinii* | Munster | M | 2004 |
| IPT124 | *B. garinii* | Guebwiller | M | 2004 |
| IPT126 | *B. garinii* | Guebwiller | M | 2004 |
| IPT128 | *B. garinii* | Guebwiller | F | 2004 |
| IPT129 | *B. garinii* | Guebwiller | F | 2004 |
| IPT130 | *B. garinii* | Guebwiller | M | 2004 |
| IPT131 | *B. garinii* | Guebwiller | M | 2004 |
| IPT133 | *B. garinii* | Guebwiller | F | 2004 |
| IPT134 | *B. garinii* | Guebwiller | F | 2004 |
| IPT136 | *B. garinii* | Guebwiller | M | 2004 |
| IPT139 | *B. garinii* | Guebwiller | F | 2004 |
| IPT140 | *B. garinii* | Munster | M | 2004 |
| IPT2 | *B. burgdorferi* s.s. | Munster | M | 2003 |
| IPT19 | *B. burgdorferi* s.s. | Guebwiller | M | 2003 |
| IPT23 | *B. burgdorferi* s.s. | Munster | F | 2003 |
| IPT24 | *B. burgdorferi* s.s. | Munster | M | 2003 |
| IPT26 | *B. burgdorferi* s.s. | Guebwiller | M | 2003 |
| IPT27 | *B. burgdorferi* s.s. | Guebwiller | M | 2003 |
| IPT35 | *B. burgdorferi* s.s. | Guebwiller | M | 2003 |
| IPT46 | *B. burgdorferi* s.s. | Munster | F | 2003 |
| IPT48 | *B. burgdorferi* s.s. | Munster | M | 2003 |
| IPT49 | *B. burgdorferi* s.s. | Munster | M | 2003 |
| IPT51 | *B. burgdorferi* s.s. | Munster | F | 2003 |
| IPT60 | *B. burgdorferi* s.s. | Guebwiller | F | 2003 |
| IPT61 | *B. burgdorferi* s.s. | Guebwiller | F | 2003 |
| IPT69 | *B. burgdorferi* s.s. | Munster | N | 2003 |
| IPT70 | *B. burgdorferi* s.s. | Guebwiller | N | 2003 |
| IPT71 | *B. burgdorferi* s.s. | Munster | N | 2003 |
| IPT77 | *B. burgdorferi* s.s. | Munster | M | 2004 |
| IPT87 | *B. burgdorferi* s.s. | Munster | F | 2004 |
| IPT92 | *B. burgdorferi* s.s. | Guebwiller | F | 2004 |
| IPT93 | *B. burgdorferi* s.s. | Munster | M | 2004 |
| IPT112 | *B. burgdorferi* s.s. | Guebwiller | M | 2004 |
| IPT125 | *B. burgdorferi* s.s. | Munster | M | 2004 |
| IPT132 | *B. burgdorferi* s.s. | Guebwiller | M | 2004 |
| IPT137 | *B. burgdorferi* s.s. | Guebwiller | F | 2004 |
| IPT141 | *B. burgdorferi* s.s. | Munster | F | 2004 |
| IPT109 | *B. afzelii* | Munster | F | 2004 |
| IPT110 | *B. afzelii* | Guebwiller | F | 2004 |
| IPT138 | *B. afzelii* | Munster | F | 2004 |
| IPT142 | *B. afzelii* | Guebwiller | M | 2004 |
