# Supplementary material for: Comparative Population Genomics of the Borrelia burgdorferi Species Complex Reveals High Degree of Genetic Isolation among Species and Underscores Benefits and Constraints to Studying Intra-Specific Epidemiological Processes
Source: PLoS One. 2014 Apr 10;9(4):e94384. doi: 10.1371/journal.pone.0094384 (PMC3993988; doi:10.1371/journal.pone.0094384)
Supplement: Table S2 — Percentage of the length of reference sequences onto which raw sequences were mapped for each strain of the B. burgdorferi species complex examined in this study. Reference sequences came from i) the chromosome (Chr), the circular plasmid cp26, and the linear plasmid lp54 of B. burgdorferi s.s. strain B31; ii) the chromosome of B. bavariensis strain PBi and the cp26 and lp54 plasmids of B. garinii strain Far04; iii) the chromosome, cp26 plasmid, and lp54 plasmid of B. afzelii strain PKo. (DOC) [file pone.0094384.s006.doc]

**Table S2. Percentage of the length of the reference sequences onto which raw sequences were mapped for each strain of the *Borrelia burgdorferi* species complex examined in this study.**

| **Strain** | ***B. burgdorferi* s.s.** | | | ***B. bavariensis*/*B. garinii*** | | | ***B. afzelii*** | | |
| --- | --- | --- | --- | --- | --- | --- | --- | --- | --- |
|  | B31 | | | PBi | Far04 | Far04 | PKo | | |
|  | Chr | cp26 | lp54 | Chr | cp26 | lp54 | Chr | cp26 | lp54 |
| IPT74 | 97.64 | 97.73 | 78.37 | 99.06 | 99.88 | 98.34 | 98.57 | 98.09 | 84.04 |
| IPT75 | 98.16 | 97.77 | 76.9 | 99.3 | 99.7 | 96.45 | 98.96 | 97.24 | 81.54 |
| IPT76 | 97.89 | 97.12 | 77.68 | 99.22 | 99.64 | 97.65 | 98.78 | 97.96 | 84.35 |
| IPT86 | 96.03 | 96.92 | 73 | 97.21 | 99.1 | 94.15 | 96.81 | 97.02 | 80.52 |
| IPT88 | 97.81 | 99.12 | 78.23 | 99.09 | 100 | 96.95 | 98.66 | 98.48 | 84.02 |
| IPT89 | 98.28 | 98.28 | 80.27 | 99.27 | 100 | 96.8 | 98.93 | 98.38 | 85.55 |
| IPT90 | 96.93 | 90.38 | 74.92 | 98.53 | 94.52 | 96.72 | 97.93 | 90.12 | 82.34 |
| IPT91 | 96.35 | 96.84 | 76.12 | 97.97 | 99.99 | 97.12 | 97.21 | 98.11 | 81.61 |
| IPT94 | 97.99 | 94.72 | 80.49 | 99.13 | 96.57 | 96.73 | 98.82 | 95.42 | 84.93 |
| IPT95 | 65.63 | 76.85 | 54.12 | 67.04 | 79.25 | 75.44 | 66.29 | 78.07 | 59.05 |
| IPT96 | 98.17 | 98.99 | 80.68 | 99.38 | 100 | 97.03 | 99.03 | 98.04 | 85.49 |
| IPT98 | 94.81 | 94.21 | 72.66 | 96.91 | 99.1 | 95.11 | 95.87 | 95.22 | 79.22 |
| IPT99 | 97.37 | 97.81 | 77.94 | 98.82 | 99.76 | 97.52 | 98.2 | 97.42 | 82.5 |
| IPT101 | 94.07 | 91.54 | 73.17 | 95.61 | 94.69 | 94.99 | 94.85 | 92.87 | 81.47 |
| IPT104 | 98.17 | 99.09 | 79.52 | 99.24 | 99.96 | 97.46 | 98.89 | 98.6 | 84.94 |
| IPT105 | 98.23 | 98.98 | 80.77 | 99.35 | 99.94 | 99.1 | 98.89 | 98.86 | 85.65 |
| IPT107 | 86.18 | 81.63 | 68.58 | 88.2 | 85.47 | 91.37 | 87.03 | 83.18 | 75.44 |
| IPT108 | 98.29 | 98.88 | 80.99 | 99.36 | 100 | 97.6 | 99.06 | 97.61 | 84.6 |
| IPT113 | 96.98 | 91.34 | 72.36 | 98.44 | 95.81 | 95.65 | 97.81 | 92.78 | 78.69 |
| IPT114 | 96.78 | 90.95 | 74.31 | 98.36 | 94.02 | 96.43 | 97.77 | 90.43 | 83.69 |
| IPT115 | 96.47 | 96.57 | 77.04 | 97.99 | 98.4 | 97.66 | 97.14 | 94.8 | 84.31 |
| IPT117 | 93.6 | 95.21 | 70.05 | 95.11 | 98.11 | 90.8 | 94.47 | 96.13 | 76.59 |
| IPT120 | 98.03 | 98.67 | 78.59 | 99.29 | 99.83 | 95.9 | 98.85 | 98.35 | 84.89 |
| IPT124 | 97.92 | 97.39 | 79.15 | 99.15 | 100 | 97.38 | 98.77 | 98.8 | 82.75 |
| IPT126 | 95.65 | 96.06 | 75.78 | 97.08 | 98.63 | 97.52 | 96.44 | 95.86 | 83.21 |
| IPT128 | 96.52 | 98.67 | 69.11 | 98.1 | 99.71 | 94.6 | 97.47 | 98.16 | 75.53 |
| IPT129 | 96.06 | 95.93 | 80.5 | 97.77 | 97.96 | 98.64 | 96.98 | 95.79 | 84.58 |
| IPT130 | 97.6 | 95.86 | 75.81 | 99 | 97.04 | 95.68 | 98.52 | 95 | 83.51 |
| IPT131 | 97.47 | 98.08 | 77.65 | 98.86 | 99.34 | 98.03 | 98.43 | 96.83 | 83.79 |
| IPT133 | 97.35 | 94.41 | 79.52 | 98.67 | 97.01 | 97.93 | 98.23 | 93.96 | 87.56 |
| IPT134 | 98.16 | 99 | 79.47 | 99.34 | 99.96 | 97.81 | 98.92 | 98.98 | 87.67 |
| IPT136 | 96.36 | 97.3 | 75.06 | 97.96 | 99.47 | 94.96 | 97.19 | 96.77 | 81.59 |
| IPT139 | 97.52 | 98.96 | 76.8 | 98.98 | 99.85 | 98.68 | 98.48 | 98.15 | 83.49 |
| IPT140 | 97.02 | 98.79 | 78.41 | 98.22 | 99.83 | 96.44 | 97.65 | 98.33 | 84.98 |
| IPT2 | 99.3 | 99.92 | 99.21 | 98.58 | 96.64 | 73.94 | 98.56 | 96.45 | 77.59 |
| IPT19 | 98.98 | 99.22 | 99.73 | 98.2 | 95.39 | 75 | 98.16 | 96.15 | 76.68 |
| IPT23 | 99.17 | 99.42 | 99.49 | 98.53 | 96.75 | 75.46 | 98.51 | 97.84 | 76.2 |
| IPT24 | 99.27 | 99.31 | 99.42 | 98.46 | 95.9 | 76.19 | 98.45 | 96.7 | 77.34 |
| IPT26 | 99.36 | 99.6 | 99.72 | 98.87 | 96.9 | 76.72 | 98.8 | 97.95 | 76.8 |
| IPT27 | 84.59 | 94.71 | 94.73 | 83.56 | 90.56 | 69.21 | 83.39 | 91.81 | 71.2 |
| IPT35 | 99.23 | 99.94 | 99.41 | 98.62 | 96.4 | 74.72 | 98.59 | 96.48 | 76.45 |
| IPT46 | 97.23 | 99.51 | 98.62 | 95.71 | 93.4 | 68.82 | 95.68 | 94.3 | 71.52 |
| IPT48 | 98.84 | 98.75 | 99.01 | 98.1 | 96.16 | 74.89 | 98 | 96.41 | 76.63 |
| IPT49 | 98.15 | 98.14 | 96.53 | 97.33 | 94.11 | 70.99 | 97.22 | 93.36 | 73.68 |
| IPT51 | 99.33 | 98.86 | 99.79 | 98.83 | 96.15 | 76 | 98.75 | 97.03 | 78.7 |
| IPT60 | 98.23 | 99.54 | 99.33 | 97.21 | 96.73 | 74.62 | 97.28 | 97.85 | 75.58 |
| IPT61 | 99.3 | 99.95 | 99.29 | 98.72 | 96.83 | 74.15 | 98.71 | 96.43 | 77.84 |
| IPT69 | 96.9 | 99.94 | 97.81 | 96.16 | 96.67 | 71.92 | 96.12 | 96.6 | 75.25 |
| IPT70 | 80.75 | 95.01 | 91.35 | 79.57 | 90.11 | 66.78 | 79.49 | 89.82 | 66.8 |
| IPT71 | 93.22 | 99.66 | 98.94 | 92.3 | 96.01 | 71.53 | 92.28 | 95.6 | 73.29 |
| IPT77 | 97.62 | 98.58 | 96.88 | 96.48 | 93.33 | 68.64 | 96.59 | 95.11 | 74.24 |
| IPT87 | 99.3 | 99.33 | 99.35 | 98.81 | 95.57 | 75.62 | 98.74 | 96.88 | 79.04 |
| IPT92 | 99.26 | 99.94 | 99.56 | 98.74 | 96.5 | 77.51 | 98.7 | 96.48 | 78.53 |
| IPT93 | 99.38 | 99.68 | 99.85 | 98.9 | 96.88 | 77.41 | 98.78 | 97.79 | 78.37 |
| IPT112 | 96.65 | 99.33 | 99.29 | 95.66 | 96.26 | 73.91 | 95.65 | 95.83 | 74.71 |
| IPT125 | 98.45 | 99.51 | 99.83 | 97.7 | 96.67 | 75.81 | 97.67 | 97.8 | 77.24 |
| IPT132 | 99.19 | 99.94 | 99.84 | 98.58 | 96.97 | 75.32 | 98.57 | 96.93 | 78.02 |
| IPT137 | 99.26 | 99.95 | 99.77 | 98.7 | 96.87 | 76.12 | 98.59 | 96.24 | 77.33 |
| IPT141 | 99.19 | 99.49 | 98.41 | 98.91 | 96.55 | 77.93 | 98.9 | 97.59 | 79.24 |
| IPT109 | 96.76 | 96.9 | 81.38 | 97.32 | 96.88 | 86.17 | 97.95 | 99.96 | 97.28 |
| IPT110 | 93.81 | 95.4 | 78.79 | 94.77 | 94.58 | 84.25 | 95.91 | 99.4 | 97.95 |
| IPT138 | 98.28 | 95.57 | 81.64 | 98.86 | 95.76 | 86.72 | 99.28 | 99.89 | 97.48 |
| IPT142 | 96.3 | 92.17 | 80.31 | 96.9 | 92.24 | 83.5 | 97.88 | 97.98 | 96.5 |
