# Supplementary material for: Comparative Population Genomics of the Borrelia burgdorferi Species Complex Reveals High Degree of Genetic Isolation among Species and Underscores Benefits and Constraints to Studying Intra-Specific Epidemiological Processes
Source: PLoS One. 2014 Apr 10;9(4):e94384. doi: 10.1371/journal.pone.0094384 (PMC3993988; doi:10.1371/journal.pone.0094384)
Supplement: Table S3 — Description of the 23 sequenced strains isolated and sequenced in previous studies and used as references in this study. (DOC) [file pone.0094384.s007.doc]

**Table S3.** **Description of the 23 sequenced strains isolated and sequenced in previous studies and used as references in this study.**

| **Strain** | **Genospecies** | **Geographical origin** | **Biological source** | **Accession Number Chr** | **Accession Number cp26** | **Accession Number lp54** | **Reference(s)** |
| --- | --- | --- | --- | --- | --- | --- | --- |
| Far04 | *B. garinii* | Denmark | Bird | ABPZ02000001-33 | CP001319 | CP001318 | [1] |
| PBr | *B. garinii* | Denmark | Human | ABJV02000001-5 | CP001305 | CP001308 | [1] |
| 94a | *B. burgdorferi* s.s. | USA (New York) | Human | ABGK02000001-9 | CP001493 | CP001500 | [2] |
| B31 | *B. burgdorferi* s.s. | USA (New York) | *I. scapularis* | AE000783 | AE000792 | AE000790 | [3] |
| Bol26 | *B. burgdorferi* s.s. | Italy | *I. ricinus* | ABCW02000001-4 | CP001568 | CP001571 | [2] |
| CA-11.2A | *B. burgdorferi* s.s. | USA (California) | *I. pacificus* | ABJY02000001-14 | CP001484 | CP001473 | [2] |
| JD1 | *B. burgdorferi* s.s. | USA (Massachusetts) | *I. scapularis* | CP002312 | CP002316 | CP001652 | [2] |
| N40 | *B. burgdorferi* s.s. | USA (New York) | *I. scapularis* | CP002228 | CP002239 | CP001651 | [2] |
| WI91-23 | *B. burgdorferi* s.s. | USA (Wisconsin) | Bird | - | CP001446 | CP001447 | [2] |
| ZS7 | *B. burgdorferi* s.s. | Germany | *I. ricinus* | CP001205 | CP001212 | CP001199 | [2] |
| 118a | *B. burgdorferi* s.s. | USA (New York) | Human | ABGI02000001-8 | CP001535 | CP001542 | [2] |
| 156a | *B. burgdorferi* s.s. | USA (New York) | Human | ABCV02000001 | CP001271 | CP001257 | [2] |
| 297 | *B. burgdorferi* s.s. | USA (Connecticut) | Human | - | CP002268 | CP001653 | [2] |
| 29805 | *B. burgdorferi* s.s. | USA (Connecticut) | *I. scapularis* | ABJX02000001-38 | CP001550 | CP001554 | [2] |
| 64b | *B. burgdorferi* s.s. | USA (New York) | Human | ABKA02000001-6 | CP001422 | CP001421 | [2] |
| 72a | *B. burgdorferi* s.s. | USA (New York) | Human | ABGJ02000001-6 | CP001375 | CP001370 | [2] |
| PKo | *B. afzelii* | Germany | Human | CP002933 | CP002934 | CP002950 | [1] |
| ACA-1 | *B. afzelii* | Sweden | Human | ABCU02000001-2 | CP001250 | CP001247 | [1] |
| PBi | *B. bavariensis* | Germany | Human | CP000013 | NC_006128 | CP000015 | [4] |
| SV1 | *B. finlandensis* | Finland | *I. ricinus* | ABJZ02000001-5 | CP001522 | CP001524 | [5] |
| VS116 | *B. valaisiana* | Switzerland | *I. ricinus* | NZ_ABCY02000001 | CP001432 | CP001433 | [6] |
| A14S | *B. spielmanii* | The Netherlands | *I. ricinus* | ABKB02000001-13 | CP001467 | CP001469 | [6] |
| DN127 | *B. bissettii* | California | *I. pacificus* | CP002746 | CP002747 | CP002761 | [6] |

1. Casjens SR, Mongodin EF, Qiu W-G, Dunn JJ, Luft BJ, et al. (2011) Whole-genome sequences of two *Borrelia afzelii* and two *Borrelia garinii* Lyme disease agent isolates. J Bacteriol 193: 6995–6996.
2. Schutzer SE, Fraser-Liggett CM, Casjens SR, Qiu W-G, Dunn JJ, et al. (2011) Whole-genome sequences of thirteen isolates of *Borrelia burgdorferi*. J Bacteriol 193: 1018–1020.
3. Fraser C, Casjens S, Huang W, Sutton GG, Clayton R, et al. (1997) Genomic sequence of a Lyme disease spirochaete, *Borrelia burgdorferi*. Nature 390.
4. Glöckner G, Lehmann R, Romualdi A, Pradella S, Schulte-Spechtel U, et al. (2004) Comparative analysis of the *Borrelia garinii* genome. Nucleic Acids Res 32: 6038–6046.
5. Casjens SR, Fraser-Liggett CM, Mongodin EF, Qiu W-G, Dunn JJ, et al. (2011) Whole genome sequence of an unusual *Borrelia burgdorferi* sensu lato isolate. J Bacteriol 193: 1489–1490.
6. Schutzer SE, Fraser-Liggett CM, Qiu W-G, Kraiczy P, Mongodin EF, et al. (2012) Whole-genome sequences of *Borrelia bissettii*, *Borrelia valaisiana*, and *Borrelia spielmanii*. J Bacteriol 194: 545–546.
