# Supplementary material for: Comparative Population Genomics of the Borrelia burgdorferi Species Complex Reveals High Degree of Genetic Isolation among Species and Underscores Benefits and Constraints to Studying Intra-Specific Epidemiological Processes
Source: PLoS One. 2014 Apr 10;9(4):e94384. doi: 10.1371/journal.pone.0094384 (PMC3993988; doi:10.1371/journal.pone.0094384)
Supplement: Table S4 — Bayes factors for each pair of coalescent models. Each cell gives the ratio of the number of simulations selected for the column model to the number selected for the row model (i.e., column: row). (DOC) [file pone.0094384.s008.doc]

**Table S4. Bayes factors for each pair of coalescent models.**

|  | M0 | M1 | M2 | M3 | M4 | M5 |
| --- | --- | --- | --- | --- | --- | --- |
| M0 | - | 0.88 | 0.54 | 1.24 | 0.83 | 0.006 |
| M1 | 1.14 | - | 0.61 | 1.40 | 0.94 | 0.007 |
| M2 | 1.86 | 1.63 | - | 2.29 | 1.54 | 0.011 |
| M3 | 0.81 | 0.71 | 0.43 | - | 0.67 | 0.005 |
| M4 | 1.21 | 1.06 | 0.65 | 1.49 | - | 0.007 |
| M5 | 167 | 147 | 90 | 206.5 | 138.5 | - |
